# A Novel Acylaminoimidazole Derivative, WN1316, Alleviates Disease Progression via Suppression of Glial Inflammation in ALS Mouse Model

**DOI:** 10.1371/journal.pone.0087728

**Published:** 2014-01-31

**Authors:** Kazunori Tanaka, Takuya Kanno, Yoshiko Yanagisawa, Kaori Yasutake, Satoshi Inoue, Noriaki Hirayama, Joh-E Ikeda

**Affiliations:** 1 NGP Biomedical Research Institute, Neugen Pharma Inc., Kitasato University School of Medicine, Sagamihara, Kanagawa, Japan; 2 Wakunaga Pharmaceutical Co. Ltd., Akitakada, Hiroshima, Japan; 3 Department of Molecular Life Sciences, Tokai University School of Medicine, Isehara, Kanagawa, Japan; 4 Department of Molecular Neurology, Kitasato University School of Medicine, Sagamihara, Kanagawa, Japan; 5 Apoptosis Research Centre, Children’s Hospital of Eastern Ontario, Department of Pediatrics, Faculty of Medicine, University of Ottawa, Ottawa, Ontario, Canada; Emory University, United States of America

## Abstract

Amyotrophic lateral sclerosis (ALS) is an adult-onset motor neuron degenerative disease. Given that oxidative stress and resulting chronic neuronal inflammation are thought to be central pathogenic, anti-oxidative agents and modulators of neuronal inflammation could be potential therapies for ALS. We report here that the novel small molecular compound, 2-[mesityl(methyl)amino]-N-[4-(pyridin-2-yl)-1H-imidazol-2-yl] acetamide trihydrochloride (WN1316) selectively suppresses oxidative stress-induced cell death and neuronal inflammation in the late-stage ALS mice. WN1316 has high blood-brain-barrier permeability and water solubility, and boosts both neuronal apoptosis inhibitory protein (NAIP) and NF-E2-related factor 2 (Nrf2) which governed glutathione (GSH)-related anti-oxidation pathway protecting motor neurons against oxidative injuries. Post-onset oral administration of low dose (1–100 µg/kg/day) WN1316 in ALS(SOD1^H46R^) and ALS(SOD1^G93A^) mice resulted in sustained improved motor function and post onset survival rate. Immunohistochemical analysis revealed less DNA oxidative damage and motor neuronal inflammation as well as repression of both microgliosis and astrocytosis, concomitant down regulation of interleukin-1β and inducible nitric oxide synthase, and preservation of the motoneurons in anterior horn of lumbar spinal cord and skeletal muscle (quadriceps femoris). Thus, WN1316 would be a novel therapeutic agent for ALS.

## Introduction

Amyotrophic lateral sclerosis (ALS) is a progressive neurodegenerative disorder characterized by a selective loss of upper and lower motor neurons, leading to death within 3–5 years. Approximately 90% of ALS cases are sporadic, while 10% of patients are familial ALS (FALS) [1]. Since Cu/Zn superoxide dismutase (*SOD1*) was originally identified as a causative gene of FALS [2], several causative genes including *FUS*, *TARDBP, OPTN*, and *ALS2* have been identified [3]. Notwithstanding these discoveries, the exact mechanism of motor neuron death in both familial and sporadic ALS is still unclear. Moreover, there remains a pressing need for an effective ALS therapy drug given the modest impact riluzole [4], [5].

Oxidative stress is being increasingly implicated in the neuronal inflammation, observed in ALS pathogenesis [6], and may possibly be a target for the development of novel therapeutic agents for ALS. The Neuronal apoptosis inhibitory protein (NAIP), which is a founding member of inhibitory of apoptosis proteins (IAPs), selectively suppresses oxidative stress-induced cell death [7], and overexpression of NAIP by adenovirus-mediated gene transfer has been shown to protect hippocampal neurons from ischemic injury [8]. Based on the endogenous NAIP functions, we previously developed NAIP-based drug screening (NAIP-ELISA) system [9], and demonstrated that the NAIP upregulating compounds exerted not only selective anti-oxidative stress activity *in vitro* [9], [10] but also *in vivo* efficacy in a transgenic ALS mouse model carrying the H46R mutation in *SOD1* gene [ALS(SOD1^H46R^)] [10], [11]. Although NAIP-ELISA system is promising strategy for the development of novel ALS treatment drugs, there is room for improvement of system in the point of optimization for time and cost.

In addition, we have recently started using in conjunction with a NAIP-ELISA system, a quantitative structure-activity relationship (QSAR) [12] used in ligand-based virtual screening [13], so that a modified algorithm for the relationship between chemical structure of NAIP upregulation compounds (selectively suppress oxidative stress induced cell death) and their anti-oxidative cell death activity *in vitro* (termed NAIP-QSAR) was applied for the compound screening *in silico*. By using the NAIP-QSAR, we screened approximately 3,260,000 commercially available compound library, and resulted in the identification of N-(4-(2-pyridyl)(1,3-thiazol-2-yl))-2-(2,4,6-trimethylphenoxy) acetamide (CPN-9), which showed a selective neuroprotection against oxidative-stress induced-cell death [14]. Moreover, the post-onset administration of CPN-9 to ALS(SOD1^H46R^) mice sustained motor functions and slowed disease progression [14]. Although CPN-9 acts as a potent remedy in ALS mice, it has also pharmacological weakness, such as a hardly water soluble, very poor blood-brain-barrier (BBB) permeability and a considerable toxicity. Thus, further modification and/or optimization of CPN-9 as a mother compound enables us to develop novel ALS therapeutic drugs.

Here we addressed this challenge with an *in silico* drug designing (termed virtual compounds), an *in silico* drug screening of virtual compounds by an algorithm of quantitative relationship between chemical structure of the compound and its anti-oxidative neuronal cell death activity (termed AOND-QSAR) [14], a chemical synthesis of virtual compounds, an *in vitro* assay of quantitative anti-oxidative neuronal cell death activity of the compounds, and an efficacy test in ALS mice.

In this study, we have successfully identified quite novel small molecular compound by performing *in silico* drug designing using CPN-9 as a primary mother compound, AOND-QSAR *in silico* screening, chemical synthesis and *in vitro* quantitative anti-oxidative neuronal cell death assay. The top hit compound was chosen as a secondary mother compound, and it was taken into *in silico* redesigning, modified AOND-QSAR screening, chemical synthesis and *in vitro* anti-oxidative neuronal cell death assay. Among hit compounds, top 4 compounds were selected, analyzed a specificity of *in vitro* anti-oxidative neuronal cell death activity, pharmacokinetics and toxicity *in vitro* as well as *in vivo*, and tested the efficacy in ALS(SOD1^H46R^) and ALS(SOD1^G93A^) mice. Among top 4 compounds, 2-[mesityl(methyl)amino]-N-[4-(pyridin-2-yl)-1H-imidazol-2-yl] acetamide trihydrochloride (WN1316) (Molecular weight: 458.82) was a compound possessing characteristics of highly soluble in water and prominent BBB permeability. *In vitro* study revealed that WN1316 exerted selectively anti-oxidative property by activating the NF-E2-related factor 2 (Nrf2)-antioxidant response element signaling pathway and upregulation of Nrf2, ATF3, p62, p21, glutathione (GSH) and NAIP.

On the other hand, to evaluate the *in vivo* efficacy of WN1316, we initiated a post onset-intragastric administration of WN1316 in ALS mice. The WN1316 administration, 1–100 µg/kg body weight/once daily per os, well sustained motor functions and prolonged the post-onset survival interval of not only ALS(SOD1^H46R^) mice but also ALS(SOD1^G93A^) mice. Further, the 10 µg/kg body weight/day WN1316 treatment ameliorated motor neuron degeneration, gliosis and oxidative damage in the anterior horn of lumbar spinal cord in ALS(SOD1^H46R^) mice. Concomitantly, inflammatory factors, interleukin-1β (IL-1β) and inducible nitric oxide synthase (iNOS), diminished dramatically. The *in vivo* effective low dose WN1316 also efficiently suppressed the axonal degeneration and muscle fiber denervation in ALS(SOD1^H46R^) mice. Besides the highly potent at low dose in ALS mice, pharmacokinetic study in non-transgenic mouse (C57BL/6N) indicated that WN1316 was highly BBB permeable compound with wide safety range. Taken together, WN1316 might represent a potential therapeutic drug for the ALS treatment.

## Results

### Identification of a Novel Acylaminoimidazole Derivative, WN1316, Selectively Suppresses Oxidative Stress Induced Cell Death

Recently, we identified a small-molecule CPN-9 which selectively suppressed oxidative stress-induced cell death via activation of the Nrf2 pathway [14]. In the search for novel ALS therapeutic agents with superior aqueous solubility, cytotoxicity and BBB permeability, further modification and/or optimization of CPN-9 as a mother compound was undertaken. We thus conducted the *in silico* design of approximately 1,390 novel molecules based on the chemical structure of a primary mother compound CPN9 which were then subjected AOND-QSAR and drug-likeness screening according to four criteria described in methods. Sixty-five molecules which best fulfill these four criteria were identified as active compounds and then broken down into 8 clusters; 3 to 4 representative virtual compounds from each cluster, 27 compounds in all, were next chemically synthesized. We then performed the anti-oxidative stress-induced neuronal cell death (AOND) analysis on these compounds, and identified a top-hit compound (WN0247) (Figure S1).

**Figure 1:**
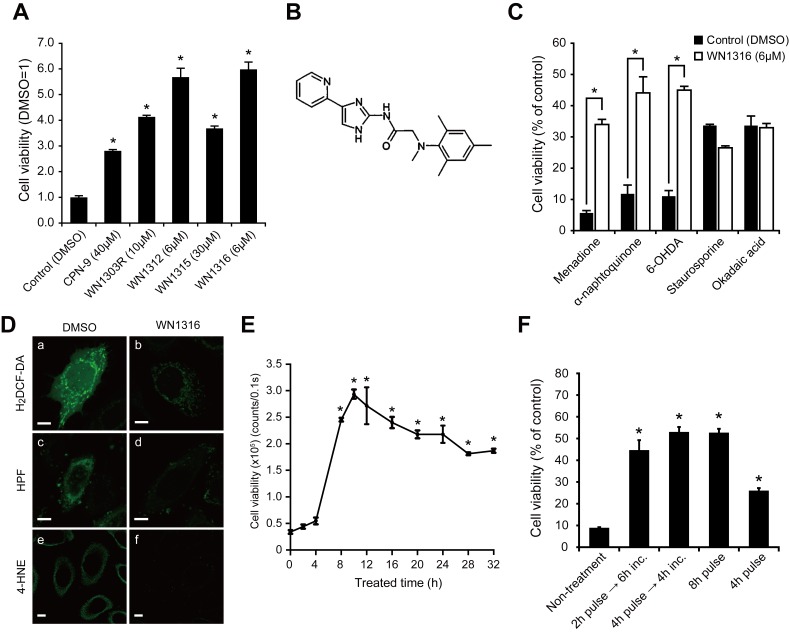
WN1316 selectively protects neuronal cells from the oxidative damage. (A) Effect of compounds against oxidative stress-induced cell death. Differentiated SH-SY5Y cells were treated with compounds or dimethyl sulfoxide (DMSO). After 24 h, cells were exposed to 60 µM menadione for 4 h. The cell viability was measured by AlamarBlue assay. The cell viability was expressed as a fold induction after the treatment with compounds compared with vehicle control (DMSO). Data are expressed as mean ± SD (n = 4). *p<0.001 by one-way ANOVA with Dunnett’s *post hoc* test compared with DMSO-treated control. (B) Chemical structure of WN1316; 2-[mesityl(methyl)amino]-N-[4-(pyridin-2-yl)-1H-imidazol-2-yl] acetamide trihydrochloride. (C) Effect of WN1316 against various cytotoxins. Differentiated SH-SY5Y cells were pretreated with 6 µM WN1316 or DMSO for 8 h at 37°C. The appropriate amount of each cytotoxin, which includes free radical generating compounds [menadione, α-naphtoquinone, 6-hydroxydopamine (6-OHDA)], protein kinase inhibitor (staurosporine), or phosphatase inhibitor (okadaic acid), was added, and incubated for another 4 to 6 h as described in Material & Methods. The cell viability was measured by AlamarBlue assay. Data are expressed as mean ± SD (n = 4). *p<0.001 by one-way ANOVA with Dunnett’s *post hoc* test compared with DMSO-treated control. (D) Representative image of the total intracellular ROS (a and b), ⋅OH (c and d), and the intracellular 4-HNE (e and f) levels in HeLa cells after oxidative insult. Cells were treated with DMSO (a, c, and e) or 10 µM WN1316 (b, d, and f) for 8 h and then loaded with 10 µM 2′,7′-dichlorodihydrofluorescein diacetate (H_2_DCF-DA) (a and b) or 100 µM hydroxyphenyl fluorescein (HPF) (c and d) for 30 min, followed by the exposure to 60 µM menadione for 1 h or 0.1 mM FeSO_4_/0.2 mM H_2_O_2_ for 1 h, respectively. For immunocytochemistry, cells were treated with DMSO or 10 µM WN1316 for 8 h, followed by the exposure to 60 µM menadione for 1 h, and immunostained with anti-4-HNE antibody (green). The fluorescent images were obtained by using a confocal microscope. Scale bars indicate 10 µm. (E) Treatment time of WN1316 on the cytoprotection. Differentiated SH-SY5Y cells were incubated with 6 µM WN1316 for the indicated times, and then treated with 60 µM menadione for 4 h. The cell viability was determined by AlamarBlue. Data are expressed as mean ± SD (n = 4). *p<0.001 by one-way ANOVA with Dunnett’s *post hoc* test compared with DMSO-treated control. (F) Treatment time of WN1316 on the cytoprotection. Differentiated SH-SY5Y cells were incubated with 6 µM WN1316 for the indicated times, and then treated with 60 µM menadione for 4 h. The cell viability was determined by AlamarBlue. Data are expressed as mean ± SD (n = 4). *p<0.001 by one-way ANOVA with Dunnett’s *post hoc* test compared with DMSO-treated control.

We again virtually designed molecules based on the chemical structure of WN0247, followed by AOND-QSAR screening, and then chemically synthesized 14 novel compounds (Figure S1). The biological activities of these compounds were tested by the AOND analysis; four active compounds, WN1303R, WN1312, WN1315 and WN1316, which showed marked cytoprotection against oxidative stress-induced cell death in neuronal SH-SY5Y cells superior to the mother compound CPN-9, were identified (Figure 1A).

To establish whether the suppression of oxidative stress-induced cell death by WN compounds was similar to the mother compound CPN-9, we evaluated their effectiveness against various cytotoxins. All showed selective protection against the oxidative stressors, menadione, α-naphthoquinone and 6-hydroxydopamine (6-OHDA), but not the non-oxidative stressors, staurosporine and okadaic acid, in differentiated SH-SY5Y cells (Figure 1C and S2). Among them, WN1316 conferred the greatest protection against oxidative stress-induced damage, and the most suppression of both reactive oxygen species (ROS) generation and lipid peroxide formation. Menadione increased the level of intracellular •OH and lipid peroxidation (Figure 1D, a,c and e) in HeLa cells, while the WN1316 treatment markedly reduced their levels (Figure 1D, b, d and f). Thus, of the four candidates, WN1316 was the most potent suppresser of highly toxic ROS generation in cultured cells.

### WN1316 is a Highly BBB Permeable Compound

We next studied the pharmacokinetics of the four WN compounds in wild type mice (C57BL/6N). To measure WN compound concentration in brain and serum (termed the area under the curve: AUC0–8), 50 mg of each WN compound in distilled water was orally administered to mouse of 8 weeks of age (n = 4). The mice tolerated the WN compounds with no apparent impact. WN1316 revealed the highest brain AUC0–8, about 39-fold of the level observed with CPN-9, and notably approximately 4-fold higher than serum levels (Table 1, Figure 1B). The clearance of WN1316 in both brain and serum was 8 h.

**Table 1:** Brain and blood concentration of four WN-compounds and CPN-9.

Compound	WN1303R	WN1312	WN1315	WN1316	CPN-9
Dose (mg/kg)	50	50	50	50	100
Number (mouse)	4	4	4	4	3
AUC_0–8_: Brain(µg⋅h/g)	13.7±5.3	16.8±3.5	3.4±0.5	38.9±5.7	1.0±1.3
AUC_0–8_: Serum(µg⋅h/ml)	9.6±3.3	13.6±3.8	3.8±0.8	13.6±0.7	5.9±0.8

Data are expressed as mean ± SD.

### WN1316 Requires Pre-conditioning Treatment for Cytoprotection against Oxidative Stress

We next assessed the temporal requirement of WN1316 for optimal *in vitro* cytoprotection, finding that pre-treatment of differentiated SH-SY5Y cells for longer than 8 h was required for AOND activity (Figure 1E). In another way, incubation of the cells with WN1316 for 2 to 4 h followed by 6 to 4 h of a chase incubation without the compound was essential to achieve a maximum cytoprotection against 60 µM menadione mediated oxidative stress induced cell death (Figure 1F). These results indicate that WN1316 activates and/or stimulates *de novo* synthesis of the endogenous proteins conferring selective protection from oxidative stress-induced damages rather than acting as a radical scavenger.

### WN1316 Induces Nrf2-regulated Gene Expression

**Figure 2:**
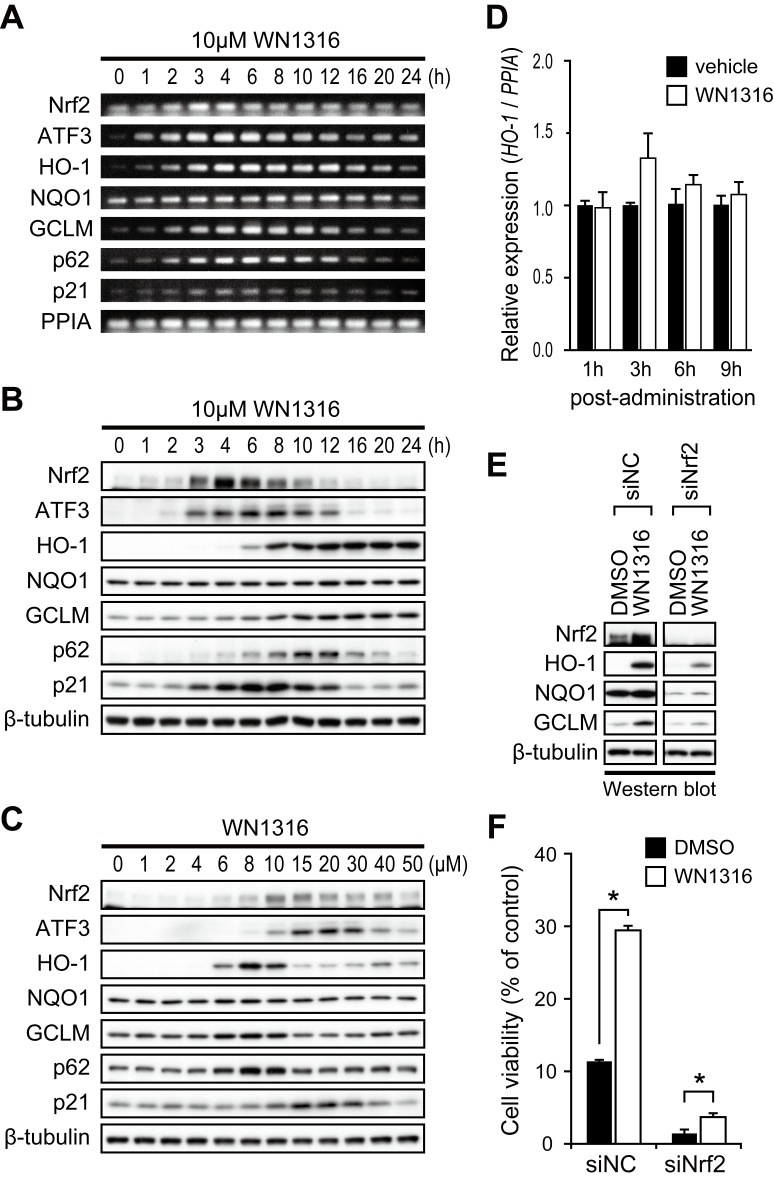
Nrf2 is essential for antioxidant and detoxifying enzyme gene induction by WN1316. (A–B) Effect of the expression of mRNAs and proteins, including Nrf2, ATF3, HO-1, NQO1, GCLM, p62 and p21, in WN1316-treated cells. Differentiated SH-SY5Y cells were treated with 10 µM WN1316 or DMSO for the indicated times. Total RNA and cell extracts from WN1316-treated SH-SY5Y cells were used for RT-PCR (A) and Western blot analysis (B), respectively. Peptidylprolyl isomerase A (PPIA) was used as a control for RT-PCR. β-tubulin was used as a loading control for protein. (C) Western blot analysis of proteins shown in A in a WN1316 dose-dependent manner. SH-SY5Y cells were incubated with 1 to 50 µM WN1316 for 8 h. Equal amount of protein from cell lysates were analyzed by using antibodies as indicated. β-tubulin was used as a loading control for protein. (D) Quantitative RT-PCR analysis of HO-1 mRNA from the lumbar spinal cord of wild type mice administered with WN1316 (100 µg/kg) (n = 3) or vehicle (physiological saline) (n = 3). Relative mRNA expression was acquired by normalizing HO-1 mRNA to PPIA mRNA. Data are expressed as mean ± SEM. (E–F) SH-SY5Y cells were transfected with 5 nM nonsilencing siRNA (siNC), or Nrf2 siRNA (siNrf2). At 48 h after transfection, cells were treated with 6 µM WN1316 for 8 h. The expression of Nrf2, HO-1, NQO1, and GCLM was analyzed by Western blotting (E). The cell viability after the exposure to 60 µM menadione for 4 h was measured by AlamarBlue and normalized to untreated control (F). Data are expressed as mean ± SD (n = 4). *p<0.001 compared with DMSO-treated control (Student’s *t*-test).

Nrf2 is a well-known ROS-activated transcription factor for anti-oxidant molecules, HO-1 and NAD(P)H dehydrogenase [quinone] 1 (NQO1) [15], [16]. Given the potent Nrf2 activation conferred by the mother compound CPN-9 [14], WN1316 was assessed for a similar effect (Figure 2A). WN1316 (10 µM) increased mRNA encoded Nrf2-regulated genes including Nrf2, ATF3, HO-1, glutamate-cysteine ligase modifier subunit (GCLM), NQO1, p21, and p62 in SH-SY5Y cells within 3 h (Figure 2A) and corresponding proteins in a time- and dose-dependent manner (Figure 2B and C). Significant nuclear translocation of cytoplasmic Nrf2 was simultaneously observed (Figure S3A). Furthermore, 100 µg/kg of oral dose of WN1316 elicited a transient induction of HO-1 mRNA gene in the lumbar spinal cord of wild-type mice 3 h post-administration (Figure 2D), suggesting that WN1316 functions *in vivo* as well as *in vitro*.

To further establish the WN1316 activation of the Nrf2 pathway, knockdown experiments of Nrf2 were conducted in SH-SY5Y cells. Nrf2-knockdown dramatically reduced HO-1, NQO1 and GCLM protein levels as well as Nrf2 protein induced by the WN1316 treatment compared to the control (Figure 2E). Further, WN1316-mediated AOND activity in SH-SY5Y cells was also reduced by Nrf2 knockdown, but not control (siNC) (Figure 2F), implicating the Nrf2 signaling pathway in WN1316-mediated cytoprotection.

### WN1316 Requires Intracellular ROS for the Activation of Nrf2 Signaling Pathway

Given that *N*-acetylcysteine (NAC), ROS scavenger, down-regulates the expression of Nrf2-regulated anti-oxidant molecule genes [14], [17], [18], we tested whether NAC moderates WN1316-mediated Nrf2 activation. NAC treatment abrogated the WN1316 induction of Nrf2, ATF3, HO-1, NQO1, GCLM, p62 and p21 mRNAs and proteins (Figure S3B and C). Concomitantly, WN1316 activity against oxidative stress-induced cell death was drastically suppressed by the NAC treatment (Figure S3D). In a second approach, modulation of NADPH oxidase, a membrane-bound enzyme, that catalyzes the production of superoxide from oxygen and NADPH [19] was undertaken. Apocynin, an NADPH oxidase inhibitor, did not affect the AOND activity of WN1316 (Figure S3D). These results suggest that the activation of Nrf2 signaling mediated by WN1316 requires intracellular ROS, but not those produced by NADPH oxidase.

Cytoplasmic Nrf2 forms a complex with Keap1 and is degraded under the basal condition, while ROS catalyzes dissociation of Nrf2 from the complex, resulting in nuclear translocation of Nrf2 [15], [16]. Although WN1316 both in the presence and absence NAC did not affect Keap1 mRNA levels, the WN1316-dependent degradation of Keap1 protein was blocked by NAC (Figure S3B and C). These results suggest that the activation of Nrf2 by WN1316 may be associated with the inhibition of Keap1-mediated Nrf2 degradation in addition to the upregulation of Nrf2 mRNA/protein.

### WN1316 Modulates GSH Levels

Since glutathione (GSH) is one of the most abundant antioxidants that scavenge reactive oxygen species, we evaluated total cellular GSH levels in differentiated SH-SY5Y cells with/without WN1316 treatment. WN1316 revealed a dose dependent AOND activity and a concomitant upregulation of total GSH level, although a higher concentration than 20 µM WN1316 militates against cytoprotection (Figure S4). These results implicate GSH modulation in WN1316 cytoprotection.

### WN1316 Upregulates NAIP

**Figure 3:**
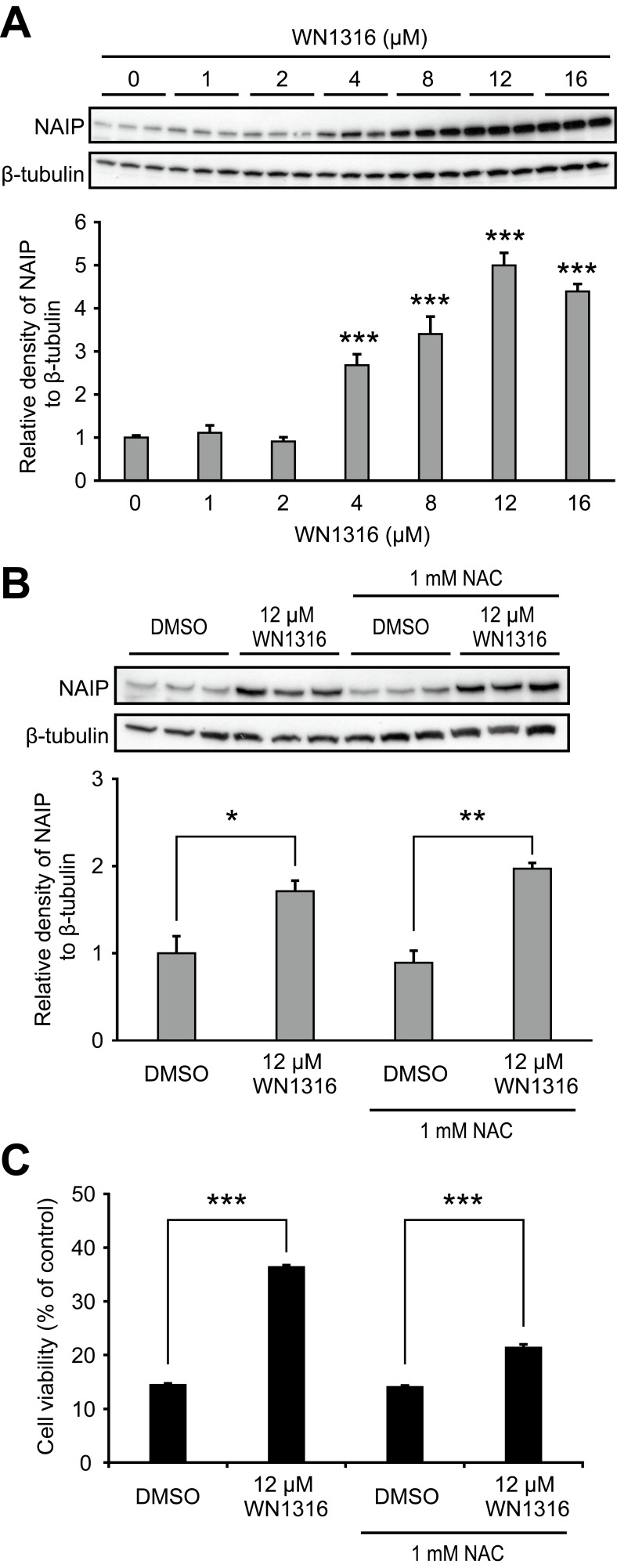
WN1316 upregulates endogenous NAIP. (A) Western blot analysis of NAIP in a WN1316 dose-dependent manner. THP-1 cells were incubated with 1 to 16 µM 1316 for 24 h. Cell lysates were analyzed with anti-NAIP antibody. β-tubulin was used as a loading control for protein. Densitometric data for NAIP bands was normalized by the level of β-tubulin. Data are expressed as mean ± SEM (n = 3). ***p<0.001 by one-way ANOVA with Dunnett’s *post hoc* test compared with untreated control. (B) Western blot analysis of NAIP in *N*-acetylcysteine (NAC)-treated THP-1 cells. Cells were incubated with 12 µM WN1316 or DMSO in the presence or absence of 1 mM NAC for 24 h. Cell lysates were analyzed anti-NAIP antibody. β-tubulin was used as a loading control for protein. Densitometric data for NAIP bands was normalized by the level of β-tubulin. Data are expressed as mean ± SEM (n = 3). * *p*<0.05 or ** *p*<0.01 by Student’s *t*-test. (C) Effect of NAC on the WN1316-mediated anti-oxidative stress activity in THP-1 cells. Cells treated with 12 µM WN1316 or DMSO in the presence or absence of 1 mM NAC were exposed to 80 µM menadione, and then the cell viability was measured by AlamarBlue. Data are expressed as mean ± SD (n = 4). *** *p*<0.0001 by Student’s *t*-test.

NAIP is a founding member of IAP family of proteins and selectively suppresses oxidative stress-induced cell death. THP-1 cells which unlike SH-SY5Y encoded an intact NAIP gene (data not shown) were exposed to WN1316. WN1316 dose-dependent upregulation of NAIP in THP-1 cells was observed and was not affected by NAC treatment (Figure 3A, B and C). Nrf2 does not impact NAIP gene transcription and biological function, suggesting that NAIP may function independently of Nrf2 in WN1316 mediated cytoprotection.

### WN1316 Slows Disease Progression in ALS(SOD1^H46R^) Mice

**Figure 4:**
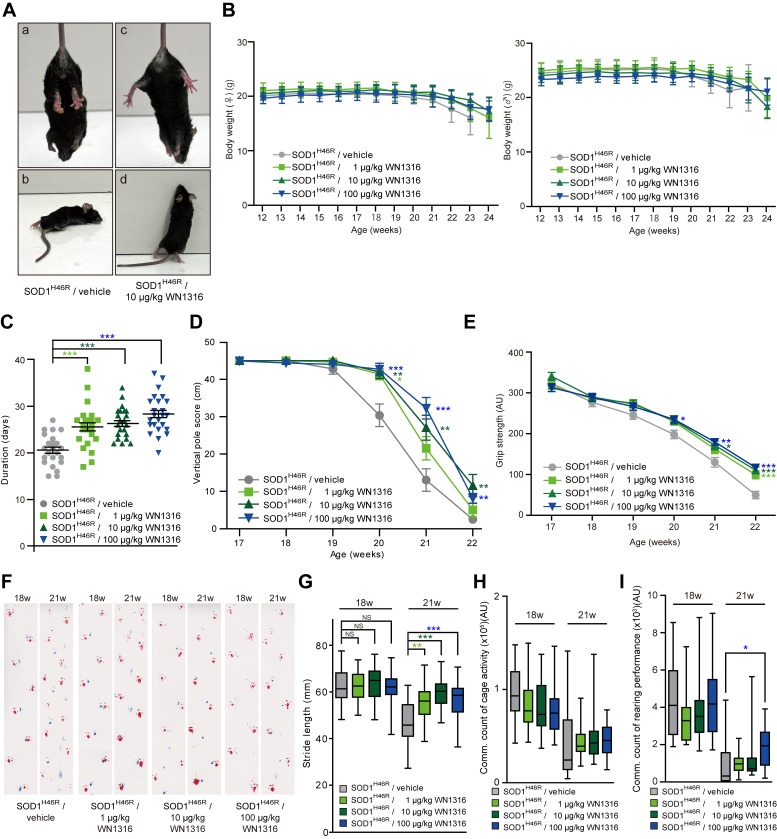
*In vivo* efficacy of WN1316 in ALS(SOD1^H46R^) mice. (A) Representative photographs of vehicle-treated (a and b) and WN1316-treated (c and d) ALS(SOD1^H46R^) mice showing a typical hind limb posture upon the tail suspension (a and c) and rearing behavior (b and d) at 157 days of age. (B) Changes in the body weight of female and male ALS(SOD1^H46R^) mice in vehicle (female, n = 13 and male, n = 13) and WN1316 [1 µg/kg, 10 µg/kg and 100 µg/kg (female, n = 13 and male, n = 13, respectively) ]-treated groups between 12 and 24 weeks of age. Data are expressed as mean ± SD. (C) Scores of the balance beam test. Duration of date from the onset to the day at which each mouse was unable to stay on the bar was plotted. Data are expressed as mean ± SEM [vehicle, 20.7±0.7 days (n = 26), 1 µg/kg WN1316, 25.6±0.9 days (n = 26), 10 µg/kg WN1316, 26.3±0.6 days (n = 26) and 100 µg/kg WN1316, 28.4±0.8 days (n = 26)]. *** *p*<0.0001 by one-way ANOVA with Bonferroni’s *post hoc* test. (D) Vertical pole scores of vehicle (n = 25) and WN1316 [1 µg/kg, 10 µg/kg and 100 µg/kg (n = 26, respectively)]-treated mice between 17 and 22 weeks of age. Data are expressed as mean ± SEM. * *p*<0.05, ** *p*<0.01 or *** *p*<0.001 by non-parametric ANOVA (Kruskal-Wallis) with Dunn’s *post hoc* test when compared to the vehicle control. (E) Grip strength of vehicle (n = 24) and WN1316 [1 µg/kg, 10 µg/kg and 100 µg/kg (n = 26, respectively)]-treated mice between 17 and 22 weeks of age. Data are expressed as mean ± SEM (AU; arbitrary unit). * *p*<0.05, ** *p*<0.01 or *** *p*<0.001 by one-way ANOVA with Bonferroni’s *post hoc* test. (F) Footprints of vehicle and WN1316 (1 µg/kg, 10 µg/kg and 100 µg/kg)-treated mice at 18 and 21 weeks of age. Blue, front paws; red, hind paws. (G) Box-whisker plots of stride length. Data indicate the average distance between the hind paw steps in vehicle (n = 21) and WN1316 [1 µg/kg, 10 µg/kg and 100 µg/kg (n = 26, respectively)]-treated mice at 18 and 21 weeks of age. * *p*<0.05, ** *p*<0.01 or *** *p*<0.001 by one-way ANOVA with Bonferroni’s *post hoc* test. NS; not significant. (H) The cage activity and (I) rearing performance of vehicle and WN1316 (1 µg/kg, 10 µg/kg and 100 µg/kg)-treated mice at 18 and 21 weeks of age. Cumulative data counting for 2 consecutive nights are shown as Box-whisker plots (AU; arbitrary unit, each group; n = 17). * *p*<0.05 by non-parametric ANOVA (Kruskal-Wallis) with Dunn’s *post hoc* test.

To assess the *in vivo* efficacy of WN1316, we conducted a daily post-onset administration of WN1316 [0 µg/kg (vehicle), 1 µg/kg, 10 µg/kg and 100 µg/kg body weight] to ALS(SOD1^H46R^) mice. The average day of disease onset was 125.1±2.4 days (n = 104). At 22 weeks of age (late symptomatic stage), a majority of the vehicle-treated mice showed a complete paralysis of hind limbs, and thus did not manifest the feet-clasping phenotype upon the tail suspension and rearing behavior (Figure 4A, a and b). In contrast, the WN1316-treated mice at the same age still showed a feet-clasping phenotype and rearing activity (Figure 4A, c and d). Despite these significant effects, WN1316 did not affect mouse weight in this study (Figure 4B).

### WN1316 Preserves Motor Performance in ALS(SOD1^H46R^) Mice

To evaluate whether WN1316 preserves motor function in ALS(SOD1^H46R^) mice, we conducted balance beam, vertical pole, grip strength and footprint tests, as well as cage activity and rearing performance analyses. The balance beam test revealed that the WN1316 (1 ∼ 100 µg/kg body weight/day) treatment significantly extended the time post onset that the mice were able to stay on the bar compared with vehicle-treated group (Figure 4C).

In contrast, there was no observable difference in the ability to perform vertical pole climbing and grip strength between the WN1316-treated and vehicle-treated groups at 17 weeks of age (late pre-symptomatic stage) (Figure 4D and E). However, at 21–22 weeks of age (late symptomatic stage), WN1316-treated groups showed a significant preservation of motor performance compared with vehicle-treated group (Figure 4D and E).

All mice at 18 weeks (early symptomatic stage) showed almost normal ambulation on footprint analysis (Figure 4F). At 21 weeks of age (late symptomatic stage), vehicle-treated mice showed an abnormal gait with reduced foot stride length and dragging of the legs, while WN1316-treated mice showed a short but still steady gait (Figure 4F and G). Furthermore, at the late symptomatic stage, the spontaneous motor activity of WN1316-treated groups was sustained in rearing performance compared with vehicle-treated group (Figure 4I), while there was a trend towards sustained cage activity in WN1316-treated groups compared with vehicle-treated group (Figure 4H).

These results demonstrate that lower doses of WN1316 (1 µg to 100 µg/kg/day) effectively sustains the motor function in ALS(SOD1^H46R^) mice and suggest that is effective *in vivo*.

### WN1316 Exerts a Beneficial Effect on Motor Function in ALS(SOD1^G93A^) Mice

Next, to investigate the *in vivo* efficacy of WN1316 in another ALS(SOD1) mouse model, ALS(SOD1^G93A^) mouse, we conducted a daily post-onset oral administration of WN1316 (10 µg/kg body weight/day) to ALS(SOD1^G93A^) mice and carried out the analysis of motor performance. The average day of disease onset was 97.7±5.3 days (n = 15). No impact of WN1316 including weight gain or loss were observed (Figure S5A). ALS(SOD1^G93A^) mice treated with WN1316 showed a sustained motor performance compared to vehicle-treated ALS(SOD1^G93A^) mice at 20 weeks of age (late symptomatic stage) by footprint analysis (Figure S5C and D). The WN1316-treated groups also showed a trend towards sustained motor performance in the balance beam test and a higher spontaneous motor activity in both cage activity and rearing performance than vehicle-treated group in a late symptomatic stage (Figure S5B, E and F). These results support the *in vivo* efficacy of WN1316 in ALS mouse models.

### WN1316 Treatment does not Alter SOD1 Expression

To ensure that the WN1316 did not alter the expression of the human *SOD1* transgene in ALS(SOD1^H46R^) mice, Western blot analysis using tissue extracts from the lumbar spinal cord in vehicle-treated and 10 µg/kg WN1316-treated groups and age-matched non-transgenic (non-Tg) littermates at 21–22 weeks of age was performed. There were no significant differences of the protein levels of mutant SOD1 in both the NP-40-soluble and the NP-40-insoluble/SDS-soluble fractions between vehicle- and WN1316-treated groups (Figure S6).

### WN1316 Represses Motor Neuron Loss in the Spinal Cord

**Figure 5:**
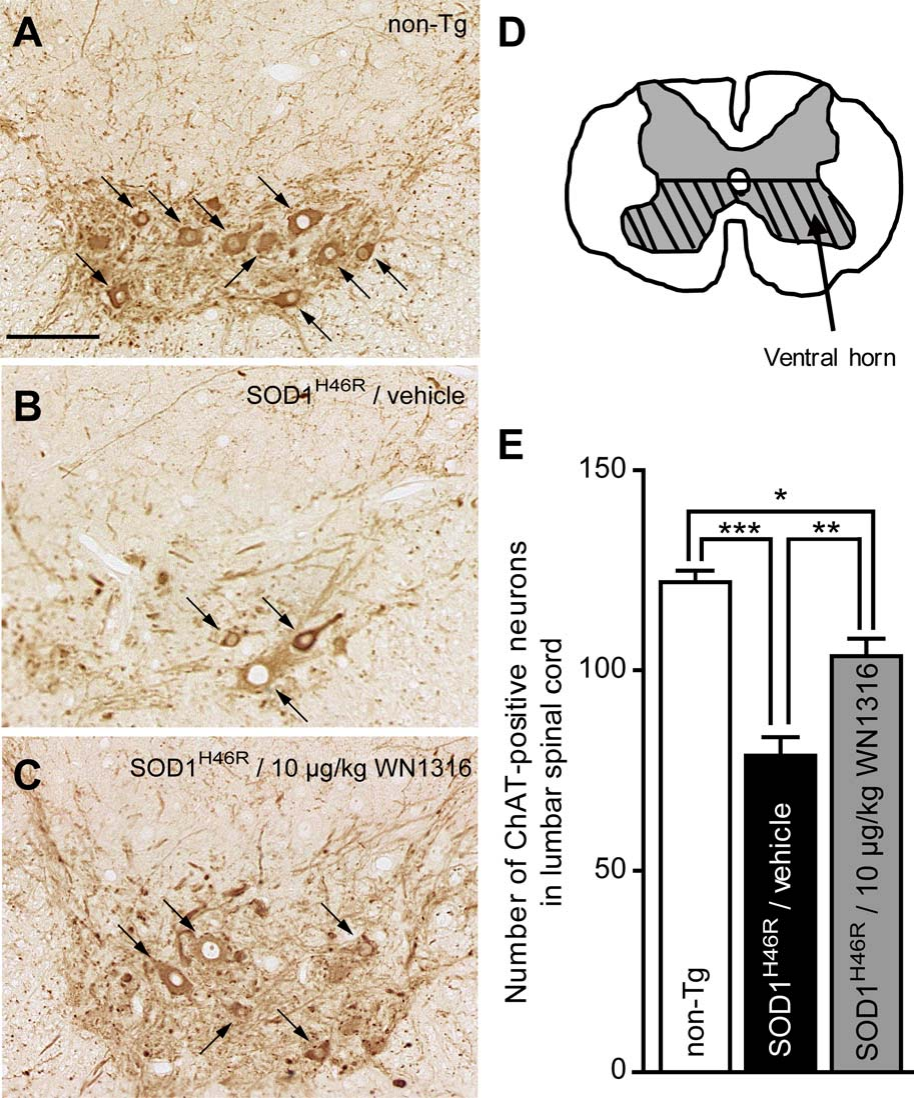
The WN1316 treatment slows motor neuron loss in the spinal cord. (A–C) Immunohistochemical analysis of paraffin embedded sections of the lumbar spinal cord (L4–L5) from non-Tg (A), vehicle-treated ALS(SOD1^H46R^) (B), and 10 µg/kg WN1316-treated ALS(SOD1^H46R^) (C) mice at 21–22 weeks of age (late symptomatic stage). Representative images of immunostaining with ChAT for the anterior horn are shown. Arrows indicate ChAT-positive motor neurons. Scale bar indicates 100 µm. (D) The number of the neurons within the hatched region of the cross-sectional L4–L5 segment in the lumbar spinal cord was counted. (E) The total number of ChAT-positive neurons (>200 µm^2^) within the ventral half of the gray matter (hatched region shown in D) in the lumbar spinal cord (L4–L5) of non-Tg (n = 4), vehicle-treated ALS(SOD1^H46R^) (n = 4), and 10 µg/kg WN1316-treated ALS(SOD1^H46R^) (n = 4) mice. Data are expressed as mean ± SEM. * *p*<0.05, ** *p*<0.01 or *** *p*<0.001 by one-way ANOVA with Bonferroni’s *post hoc* test.

To investigate whether WN1316 impacts motor neuron counts, lumbar sections (L4–L5) were immunostained using anti-choline acetyltransferase (ChAT) (for motor neurons) antibody. At 21–22 weeks of age, the vehicle-treated ALS(SOD1^H46R^) mouse showed a massive loss of ChAT-positive motor neurons (Figure 5B) compared with non-Tg littermates (Figure 5A), whereas ChAT-positive motor neurons of 10 µg/kg WN1316-treated ALS(SOD1^H46R^) mouse were relatively spared (Figure 5C). The number of ChAT-positive motor neurons within the hatched region shown in Figure 5D was counted. At 21–22 weeks of age, the loss of ChAT-positive motor neurons was 35.5% in vehicle-treated and 15.2% in WN1316-treated ALS(SOD1^H46R^) mice when compared with non-Tg littermates (Figure 5E) consistent with sparing of the motor neurons.

### WN1316 Prevents the Activation of Microglia and Astrocytes in Spinal Cord

**Figure 6:**
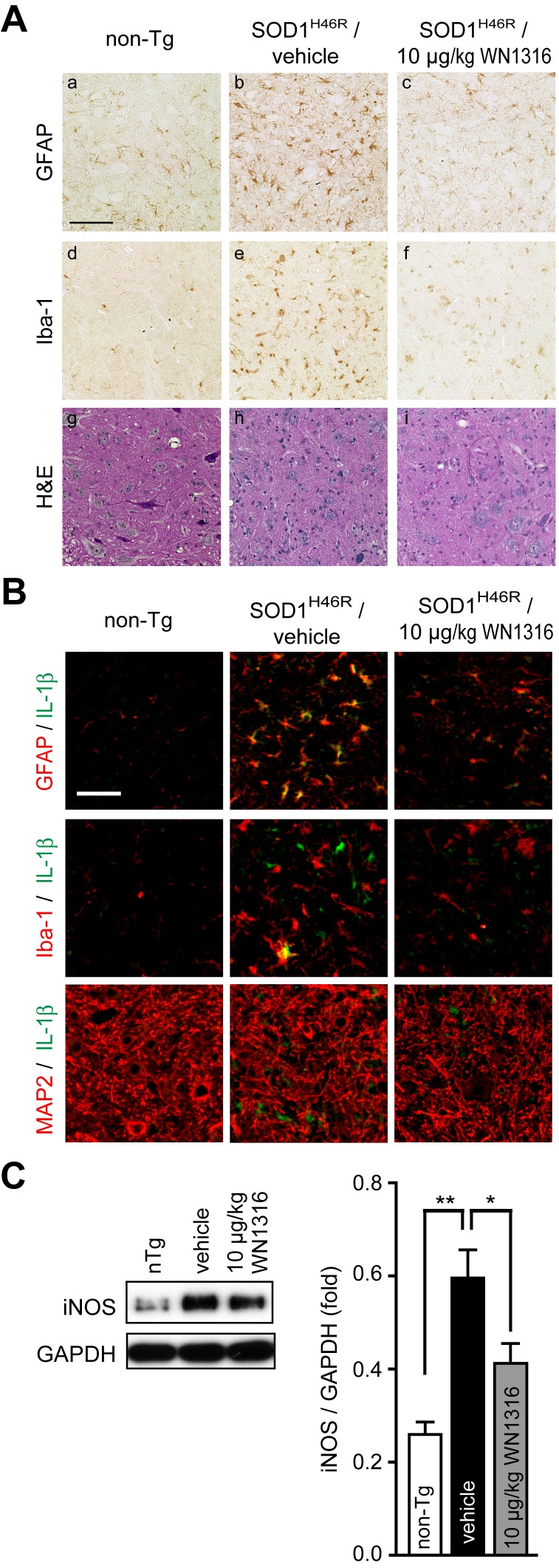
The WN1316 treatment suppresses astrocytic and microglial activation and reduces the level of IL-1β and iNOS in the spinal cord. (A) Immunohistochemical analysis of paraffin embedded sections of the lumbar spinal cord from non-Tg (a, d, and g), vehicle-treated ALS(SOD1^H46R^) (b, e, and h), and 10 µg/kg WN1316-treated ALS(SOD1^H46R^) (c, f, and i) mice at 21–22 weeks of age (late symptomatic stage). Representative images of immunostaining with GFAP (a, b, and c) and Iba-1 (d, e, and f) and histochemical staining with hematoxylin-eosin (H&E) (g, h, and i) are shown. Scale bar indicates 100 µm. (B) Immunohistochemical analysis of frozen sections of the lumbar spinal cord from non-Tg, vehicle-treated ALS(SOD1^H46R^), and 10 µg/kg WN1316-treated ALS(SOD1^H46R^) mice at 21–22 weeks of age (late symptomatic stage). Representative merged images of double immunostaining with IL-1β (green) and GFAP (for astrocytes) (red), Iba-1 (for microglia) (red), or microtubule-associated protein 2 (MAP2) (for neurons) (red) are shown. Merged signals are shown in yellow. Scale bar indicates 50 µm. (C) Western blot analysis of the iNOS protein in the lumbar spinal cord from ALS(SOD1^H46R^) mice treated with vehicle or 10 µg/kg WN1316 at 21–22 weeks of age (late symptomatic stage) and from age-matched non-Tg littermates, and quantitative analysis of iNOS-immunoreactive bands. The NP-40-soluble fraction (2 µg) was used for immunoblotting with anti-iNOS antibody. Glyceraldhyde-3-phosphate dehydrogenase (GAPDH) was used as control for NP-40-soluble fraction. Densitometric data for iNOS-immunoreactive bands was normalized by the level of GAPDH. Data are expressed as mean ± SEM (n = 4). * *p*<0.05, ** *p*<0.01 by one-way ANOVA with Tukey’s *post hoc* test.

Spinal cord glial cells activation is a histopathological hallmark both of human and murine ALS [20], [21], [22]. To analyze the impact of WN1316 on glial cell activation at sites of motor neuron loss in ALS(SOD1^H46R^) mice, immunostaining with anti-glial fibrillary acidic protein (GFAP) (for astrocytes) and anti-ionized calcium binding adaptor molecule 1 (Iba-1) (for activated microglia) antibodies using lumbar sections (L4–L5) was performed. At 21–22 weeks of age, marked immunoreactivities both for GFAP and Iba-1 were observed in lumbar spinal cord anterior horn of vehicle-treated ALS(SOD1^H46R^) mice (Figure 6A, b and e), but not age-matched non-Tg (Figure 6A, a and d). WN1316 (10 µg/kg)-treated mice exhibited significant reduction of the GFAP and Iba-1 immunoreactivity (Figure 6A, c and f) and preservation of anterior horn cells (Figure 6A, i) compared with vehicle-treated mice (Figure 6A, h). These results reveal that the WN1316 alleviates both astrocytosis and microgliosis in the spinal cord of ALS(SOD1^H46R^) mice in the late symptomatic stage.

### WN1316 Suppresses the Production of Inflammatory Factors

To examine whether WN1316 modulates the production of proinflammatory mediator IL-1β and inflammatory marker iNOS, we performed immunohistostaining for IL-1β and Western blotting for iNOS. A marked IL-1β immunoreactivity was detected in astrocytes and partially microglia, but not in neuron, in lumbar spinal cord anterior horn of vehicle-treated ALS(SOD1^H46R^) mice at 21–22 weeks of age compared to non-Tg littermates (Figure 6B). Importantly, 10 µg/kg WN1316-treated ALS(SOD1^H46R^) mice revealed that the IL-1β immunoreactivity diminished dramatically in astrocytes (Figure 6B).

We also detected an increased level of iNOS in the extract from lumbar spinal cord of vehicle-treated ALS(SOD1^H46R^) mice at 21–22 weeks of age compared to those in non-Tg littermates (Figure 6C), whereas the WN1316 treatment decreased iNOS generation in ALS(SOD1^H46R^) mice (Figure 6C).

### WN1316 Reduces Oxidative Damage in ALS(SOD1^H46R^) Mice

**Figure 7:**
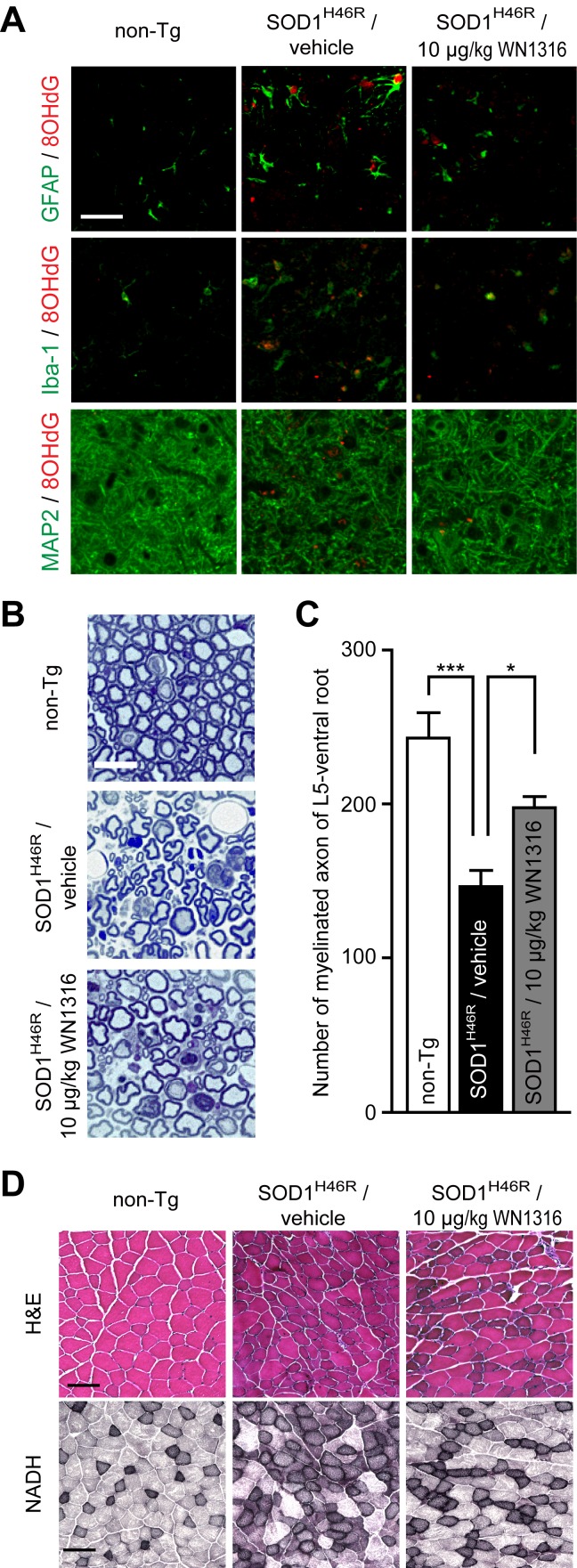
The WN1316 treatment mitigates oxidative damage to DNA and degeneration of axon and muscle. (A) Immunohistochemical analysis of paraffin embedded sections of the lumbar spinal cord from non-Tg, vehicle-treated ALS(SOD1^H46R^), and 10 µg/kg WN1316-treated ALS(SOD1^H46R^) mice at 21–22 weeks of age (late symptomatic stage). Representative merged images of double immunstaining with 8OHdG (red) and GFAP (for astrocytes) (green), Iba-1 (for microglia) (green), or MAP2 (for neurons) (green) are shown. Scale bar indicates 50 µm. (B) Representative toluidine blue staining of the transverse section of L5 ventral root from non-Tg, vehicle-treated ALS(SOD1^H46R^), and 10 µg/kg WN1316-treated ALS(SOD1^H46R^) mice at 21–22 weeks of age (late symptomatic stage). Scale bar indicates 20 µm. (C) Quantitative analysis of large myelinated axon numbers in L5 ventral root. Data are expressed as mean ± SEM (n = 4). * *p*<0.05, *** *p*<0.001 by one-way ANOVA with Bonferroni’s *post hoc* test. (D) H&E and NADH staining of quadriceps femoris muscle from non-Tg, vehicle-treated ALS(SOD1^H46R^), and 10 µg/kg WN1316-treated ALS(SOD1^H46R^) mice at 21–22 weeks of age (late symptomatic stage). Scale bar indicates 100 µm.

DNA oxidative damage has been found in the spinal cord of ALS patients [23]. To test whether the WN1316 treatment suppresses oxidative damage to DNA, we immunostained lumbar sections (L4–L5) of ALS(SOD1^H46R^) mice using anti-8-hydroxy-2-deoxyguanosine (8-OHdG) antibody. At 21–22 weeks of age, DNA oxidation was detected in among neurons, astrocytes and microglia of the lumbar spinal cord in vehicle-treated ALS(SOD1^H46R^) mice compared to non-Tg littermates (Figure 7A). Notably, 10 µg/kg WN1316-treated ALS(SOD1^H46R^) mice exhibited a remarkable reduction of DNA oxidation (Figure 7A).

### WN1316 Mitigates the Ventral Motor Neuroaxonal Degeneration in ALS(SOD1^H46R^) Mice

It has been suggested that oxidative stress plays a major role in axonal degeneration [24]. To assess the effect of WN1316 on axonal degeneration, we counted large myelinated axons in the L5 ventral root. At 21–22 weeks of age, vehicle-treated ALS(SOD1^H46R^) mice showed a marked loss of large myelinated axons compared to non-Tg littermates, while 10 µg/kg WN1316-treated ALS(SOD1^H46R^) mice showed the preservation of large myelinated axons (Figure 7B and C).

### WN1316 Reduces Myofibrosis and Myonecrosis of the Skeletal Muscle in ALS(SOD1^H46R^) Mice

To evaluate the effect of WN1316 on skeletal muscle integrity, the morphology of muscle fibers in quadriceps femoris muscle was examined. Transverse quadriceps femoris muscle of vehicle-treated ALS(SOD1^H46R^) mice at 21–22 weeks of age demonstrated a severe abnormal pattern of fiber distribution and fiber type grouping (myonecrosis and myofibrosis) compared with age-matched non-Tg mouse (Figure 7D). However, the WN1316 treatment preserved the skeletal muscle integrity in the ALS(SOD1^H46R^) mice (Figure 7D).

### WN1316 Prolongs a Post-onset Survival Interval of ALS Mice

**Figure 8:**
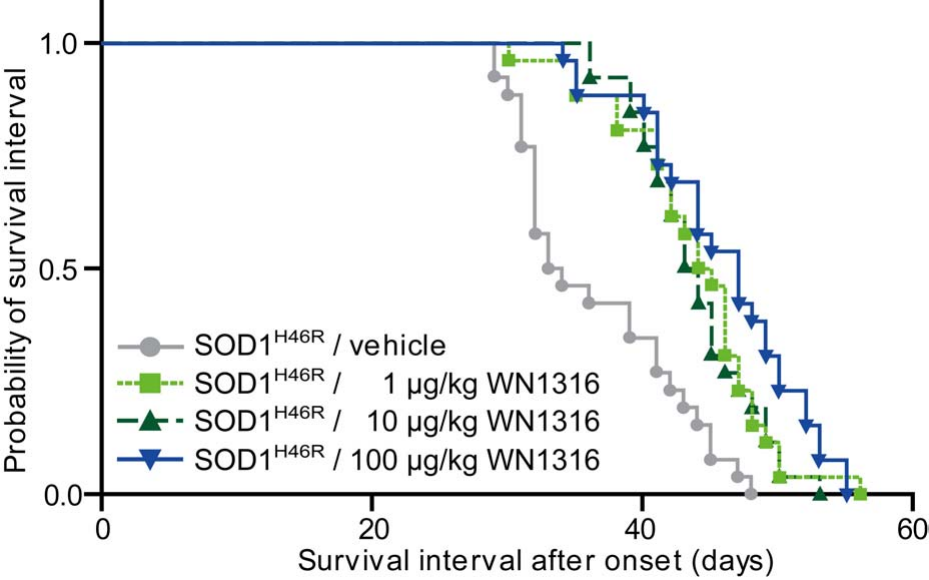
The WN1316 treatment prolongs the survival interval after onset in ALS(SOD1^H46R^) mice. The Kaplan-Meier curves demonstrate the probability of survival interval of vehicle control and WN1316 (1 µg/kg, 10 µg/kg and 100 µg/kg)-treated ALS(SOD1^H46R^) mice. The average onset of ALS(SOD1^H46R^) mice was 125.1±2.4 days (n = 104). Survival intervals in WN1316-treated groups at doses of 1 µg/kg (43.8±5.5 days, n = 26), 10 µg/kg (43.9±4.4 days, n = 26) and 100 µg/kg (45.9±6.0 days, n = 26) were significantly longer than that in vehicle group (36.6±6.2 days, n = 26) (*p*<0.05 by log-rank test). These data are expressed as mean ± SD.

We finally investigated the effect of WN1316 on disease progression and survival in ALS(SOD1^H46R^) mice. Kaplan-Meier survival analysis revealed that the survival intervals after onset in WN1316-treated animals with a dose of 1 µg/kg (43.8±5.5 days, n = 26), 10 µg/kg (43.9±4.4 days, n = 26) and 100 µg/kg (45.9±6.0 days, n = 26) were extended in comparison with those in vehicle group (36.6±6.2 days, n = 26), respectively (Figure 8).

Further, ALS(SOD1^G93A^) mice treated with WN1316 showed prolonged post-onset life span (66.1±12.0 days, n = 8) in comparison with mice treated with vehicle (57.9±6.0 days, n = 7) (Figure S5G).

These results demonstrate that WN1316 alleviates disease progression in the ALS mouse models.

## Discussion

Although the molecular mechanism of selective motor neuron loss in ALS is still unclear, substantial evidence points towards oxidative stress and neuronal inflammation playing a crucial role in ALS pathogenesis [6]. Employing NAIP-ELISA [9] and NAIP-QSAR [14], we have previously identified CPN-9 as potential novel ALS therapeutic agent. To overcome CPN-9’s innate pharmacological weakness (e.g. poor solubility, poor BBB permeability and cytotoxicity), we used it as a mother compound to identify WN1316 as a first-in-class potential ALS therapy.

Previously, we showed that CPN-9 mediated the activation of Nrf2 signaling cascade [14]. In general, Nrf2 defends the cells against oxidative stress-induced cell death [25], [26] by dissociation of cytoplasmic Nrf2-Keap 1 complexes followed by nuclear translocation of Nrf2 [25], [26], [27], [28], [29], In this study, we have demonstrated that WN1316 acts as Keap1 inhibitor and selectively suppresses oxidative stress-induced cell death via the activation of the Nrf2 signaling pathway. WN1316 also significantly increased both mRNA and protein levels of Nrf2-related genes including p21 and p62 with attendant increased cell viability. In addition, the generation of mitochondrial ROS (mitROS) is also associated with the activation and stabilization of Nrf2 [30]. We also showed that WN1316 exerted cytoprotective activity against oxidative injury through the activation of Nrf2 signaling pathway in a mitROS-dependent but not NADPH oxidase-derived ROS-dependent manner. In addition, it has been reported that Cu^2+^ enhances induction of HO-1 by 2-tert-butyl-1,4-hydroquinone (tBHQ), which is a known inducer of Nrf2 [31]. In the present study, we demonstrated that WN1316 activity *in vitro* required Cu^2+^ (data not shown).

WN1316 also upregulates NAIP independently from Nrf2 activation pathway (Figure S7). Mitochondrial, not cytoplasmic, Ca^2+^ stimulates NAIP upregulation (data not shown). Thus, WN1316 primarily affects mitROS and Ca^2+^ generation and achieves a selective protection against oxidative injury by a complementary and parallel courses of NAIP- and Nrf2-anti-oxidation pathways *in vitro* (Figure S7).

To date, there are no effective therapeutic interventions that markedly improve the quality of life for ALS patients. In this study, we sought to evaluate the *in vivo* efficacy of WN1316 in both ALS(SOD1^H46R^) and ALS(SOD1^G93A^) mice. WN1316 did not affect weight in mice (Figure 4B). Autopsy of WN1316-treated mice also exhibited no pathological abnormalities of organs such as heart and kidney (data not shown), indicating that the doses of WN1316 in our study are not harmful to mice. We have demonstrated that the post-onset administration of WN1316 preserved motor performance and improved post-onset survival rate, 20–25% prolongation in ALS(SOD1^H46R^) and 14% prolongation in ALS(SOD1^G93A^) mice. The WN1316 treatment also delayed motor neuron loss in the spinal cord accompanied by the suppression of glial cells activation in the late symptomatic stage ALS(SOD1^H46R^) mice. Further, WN1316 transiently upregulates an mRNA level of Nrf2-related gene HO-1 in a wild type mouse spinal cord, implying WN1316 actions *in vivo* are analogous to those observed *in vitro*. Recent studies have suggested that Nrf2-dependent oxidative cytoprotection is mediated in part by ATF3 modulation of GSH levels [32], and that GSH suppresses the production of proinflammatory factors [33]. Indeed, we have demonstrated that the WN1316 treatment augmented the level of ATF3 protein and GSH in human neuronal cells *in vitro* and down-regulated the level of IL-1β and iNOS in the lumbar spinal cord of ALS(SOD1^H46R^) mice. Moreover, we have confirmed NAIP, which suppresses neuronal cell death induced by oxidative injury [9], is upregulated in WN1316-treated cells *in vitro*. Thus, it is conceivable that WN1316 protects from motor neuron loss via the combined pathway involving the enhancement of NAIP- and Nrf2-mediated cytoprotection and the intracellular GSH augmentation in ALS(SOD1^H46R^) mice (Figure S7).

Inflammation in ALS is characterized by the massive activation of astrocytes and microglia [34], [35] elevating the production of inflammatory markers and proinflammatory cytokines [36], [37]. Several lines of evidence have reported that astrocytes have an impact on motor neuron degeneration [38], [39] and microglia contributes to neuronal damage in neurodegenerative diseases [37], [40]. Further, the link between oxidative stress and inflammatory response in ALS has been suggested [6], [41]. In this study, we demonstrated that WN1316 suppressed both microgliosis and astrocytosis while concomitantly curbing IL-1β and iNOS generation in the spinal cord of ALS(SOD1^H46R^) mice. In addition, the WN1316 reduced the level of DNA oxidation in neurons, astrocytes and microglia of the spinal cord in the late symptomatic stage ALS(SOD1^H46R^) mice. Although the molecular base of the WN1316 effect on astrocytes and microglia remains elusive, WN1316 attenuates a gliosis and motoneuronal inflammation by inhibiting the production of inflammatory factors, resulting in the reduction of oxidative damage in the spinal cord and the rescue of motor neurons and ventral motor axons.

There are many reports of small compounds that show an *in vivo* effect with measure of concentration in the animal studies of ALS [42], [43]. In our study, the effective *in vivo* oral dose of WN1316 is extremely low ranging 1 ∼ 100 µg/kg/day. WN1316 concentration in brain and blood of non-transgenic control C57BL/6N mouse (oral administration of 50 mg/kg body weight) at the point of Cmax/AUC0–8 were 42.1 µM/38.9±5.7 µg⋅h/g and 9.78 µM/13.6±0.7 µg⋅h/ml, respectively. It is obvious that WN1316 is highly BBB permeable and biologically active compound, despite the fact that WN1316 in rat/human sera complexes with protein (unpublished data). Oddly, it appears that the *in vitro* effective WN1316 concentration is higher than the *in vivo* case. The difference in effective concentration between *in vitro* and *in vivo* studies may reflect chaperon-mediated WN1316 transport (Figure S7), the identification of which is currently underway.

The development of novel and effective ALS drugs requires testing in animal models and pathological studies. Guidelines for the preclinical animal study in ALS recommends administration of candidate agents at symptomatic phase as is the case in the clinical setting [44]. In accordance with this guideline, we conducted post-onset oral administration of WN1316 to SOD1 ALS mouse model, demonstrating that WN1316 remarkably ameliorated clinical symptoms. To further verify the WN1316 efficacy, it might be ideals that WN1316 is tested in another ALS models besides SOD1 mutations, but any animal or cell models suit to our experimental condition are hardly available at this moment. However, it is noteworthy that WN1316 shows analogous *in vivo* effect in different lines of ALS mouse model, ALS(SOD1^H46R^) and ALS(SOD1^G93A^) mice. That is, our results prove the drug efficacy of WN1316 in the preclinical animal study. Although the mechanism of WN1316 action is not yet fully understood, WN1316 is a promising candidate as a novel therapeutic agent for the treatment of ALS. The preclinical safety tests and chemistry, manufacturing and control (CMC) of WN1316, and a drug formulation for Phase1 clinical test are presently ongoing.

## Materials and Methods

### Chemicals and Materials

CPN-9 was synthesized by Shinsei Chemical Company Ltd. (Japan) (Kanno, et al. 2012). WN-compounds were synthesized by Wakunaga Pharmaceutical Co., Ltd. (Japan). The purity of the compounds is higher than 99.2%.

We purchased AlamarBlue, MitoSOX Red and TRIzol reagent from Invitrogen, 2′,7′-dichlorodihydrofluorescein diacetate (H_2_DCF-DA), 6-hydroxydopamine (6-OHDA), α-naphthoquinone and menadione from Sigma (St. Louis), hydroxyphenyl fluorescein (HPF) from Sekisui Medical Co., Ltd. (Japan), staurosporine from Alexis Corporation (San Diego), *all*-*trans* retinoic acid, culture media (DMEM), and okadaic acid from Wako Pure Chemical Industries, Ltd. (Japan). General laboratory reagents were obtained from Nacalai Tesque (Japan) and Sigma (St. Louis). All other laboratory reagents are from commercial sources and of analytical grade.

### Antibodies

Primary antibodies used for Western blotting included rabbit polyclonal anti-SOD1 (1∶20,000, Santa Cruz Biotechnology), rabbit polyclonal anti-NOS2 (iNOS) (M-19) (1∶1000, Santa Cruz Biotechnology), anti-Nrf2 (EP1808Y) (1∶2000, EPITOMICS), anti-ATF3 (C-19) (1∶1000, Santa Cruz Biotechnology), anti-HO-1 (H105) (1∶1000, Santa Cruz Biotechnology), anti-NQO1 (A180) (1∶1000, Santa Cruz Biotechnology), anti-GCLM (FL-274) (1∶1000, Santa Cruz Biotechnology), anti-p21 (12D1) (1∶1000, Cell Signaling Technology), anti-p62 (GP62-C) (1∶10,000, Progen), anti-lamin B1 (1∶10,000, Abcam) and anti-β-tubulin (1∶20,000, Sigma), anti-glyceraldhyde-3-phosphate dehydrogenase (GAPDH) mouse monoclonal (1∶10,000, CHEMICON), anti-β-actin rabbit polyclonal (1∶1000, Cell Signaling Technology) antibodies. Anti-NAIP rabbit antiserum was raised by immunizing Japanese White rabbits with full-length NAIP protein. Secondary antibodies used in this study included ECL™ anti-rabbit IgG, Horseradish Peroxidase linked whole antibody (1∶5000, Amersham Biosciences) and ECL™ anti-mouse IgG, Horseradish Peroxidase linked whole antibody (1∶5000, Amersham Biosciences) for Western blotting.

For primary antibodies for immunohistochemistry, rabbit polyclonal anti-Iba-1 (1∶200, Wako), rabbit polyclonal anti-GFAP (1∶200, Lab Vision), goat anti-ChAT polyclonal (1∶100, CHEMICON), goat polyclonal anti-IL-1β (1∶50, R&D systems), mouse monoclonal anti-8-OHdG (2 µg/ml, NIKKEN SEIL Co., Ltd.) and rabbit polyclonal anti-microtubule-associated protein 2 (MAP2) (1∶1000, Abcam) antibodies were used. For double-immunostaining with anti-8-OHdG antibody, anti-GFAP, anti-Iba-1 and MAP2 antibodies were used. For double-immunostaining with anti-IL-1β antibodiy, anti-GFAP (1∶1000, Lab Vision), anti-Iba-1 (1∶1000, Wako) and MAP2 (1∶2000, Abcam) antibodies were used.

### *In silico* Screening

*In silico* screening was applied to screen the virtual molecules designed *in silico*. A group of drugs with similar pharmacological function usually share common chemical characteristics. *In silico* screening was configured employing the following four criteria; 1) Quantitative structure-activity relationship (QSAR) equation derived using the chemical structure and the biological (anti-oxidative stress-induced neuronal cell death: AOND) activities of active molecules (AOND-QSAR) [14], 2) CNS drug-likeness which are specific chemical properties shared among drugs acting upon the central nervous system (CNS), 3) presumptive blood-brain barrier permeability, and 4) pharmacophoric analysis comprised of an ensemble of steric and electronic features to ensure the optimal supramolecular interaction with a specific biological target and to trigger its biological response. A number of compounds were synthesized employing these criteria, and assessed for activity in an AOND assay.

*In silico* virtual molecule designing 1^st^ novel hit compound and the *in silico* screening as stated above, and AOND analysis were conducted. All calculations were performed by the use of the software system MOE (Molecular Operating Environment; Chemical Computing Group Inc., Montreal, Quebec, Canada).

### Anti-oxidative Stress-induced Neuronal Cell Death (AOND) Analysis

Human neuroblastoma SH-SY5Y cells (ATCC, CRL-2266) were grown in Dulbecco’s modified Eagle’s medium (DMEM) supplemented with 10% fetal bovine serum (HyClone, Thermo Fisher Scientific Inc.), 100 U/ml penicillin, and 100 µg/ml streptomycin at 37°C in 5% CO_2_. For differentiation, SH-SY5Y cells were seeded at a density of 0.75×10^4^ cells per well in 96-well plates (Primaria, BD Falcon) for 1 day, and cultured in the medium containing 10 µM all-trans retinoic acid for additional 5 days.

SH-SY5Y cells were plated at density of 0.75×10^4^ cells/well in 96-well plates and treated with various concentrations of compound dissolved in dimethyl sulfoxide (DMSO) or vehicle control (DMSO), and cultured for another 24 h. Then, the cells were exposed to various concentrations of cytotoxins, including oxidative stressors [menadione, 10 to 80 µM for 4 to 6 h; α-naphthoquinone, 12.5 µM for 4 h; 6-hydroxydopamine (6-OHDA), 400 µM for 4 h], protein kinase inhibitor (staurosporine, 25 µM for 6 h) and phosphatase inhibitor (okadaic acid, 1 µM for 6 h), in fresh medium. Cell viability was measured by AlamarBlue assay (Invitrogen) as described previously [9].

### Detection of Intracellular ROS

HeLa cells (ATCC, CCL-2) cultured on poly-L-lysine-coated coverslips were treated with WN1316 (10 µM) or vehicle (DMSO) for 8 h and incubated with 10 µM H_2_DCF-DA for 30 min. Cells were then exposed to 60 µM menadione for 1 h. Confocal fluorescence images were obtained using Zeiss LSM 700 confocal microscope.

To detect the hydroxyl radical (•OH), we used hydroxyphenyl fluorescein (HPF) as a specific probe. In brief, HeLa cells were treated with WN1316 (10 µM) or vehicle (DMSO) for 8 h and then incubated with 100 µM HPF for 30 min at 37°C in Hanks’s buffer (Invitrogen). After washing with phosphate-buffered saline (PBS), the reaction buffer (0.1 mM FeSO_4_, 0.2 mM H_2_O_2_) was added, and cells were incubated for 1 h at 37°C to generate •OH. Confocal fluorescence images were obtained using the lambda mode of Zeiss LSM 510 Meta confocal microscope and analytical software.

### Immunocytochemistry

HeLa cells cultured on poly-L-lysine-coated coverslips were treated with 10 µM WN1316 or DMSO for 8 h, and then incubated in fresh medium containing 60 µM menadione for 1 h. Cells were fixed in 0.1% glutaraldehyde/4% paraformaldehyde/PBS for 10 min, rinsed three times with PBS, and permeabilized with 0.1% Triton-X-100 for 15 min. Specimens were blocked with 5% normal goat serum/1% bovine serum albumin/0.1% Triton-X-100/PBS and incubated with 4-HNE monoclonal antibody (HNEJ-2) (1∶10, JaICA) as a marker of radical-induced lipid peroxidation. A goat anti-mouse IgG antibody-conjugated CF488A (#20018) (1∶400, Biotium) was used for immunofluorescent imaging. Cells were visualized using the lambda mode of a Zeiss LSM 510 Meta confocal microscope and analytical software.

### Reverse Transcriptase-polymerase Chain Reaction (RT-PCR)

Total RNA was extracted from cells using the TRIzol reagent (Invitrogen). Isolated total RNA was purified using the SV Total RNA Isolation System (Promega) according to the manufacturer’s instruction. RT-PCR was performed with QIAGEN onestep RT-PCR kit (QIAGEN). The housekeeping peptidylprolyl isomerase A (PPIA) gene was used as an internal control. PCR primers specific to each gene are as follows: ATF3, 5′-CTCCTGGGTCACTGGTGTTT-3′, and 5′-AGGCACTCCGTCTTCTCCTT-3′ (268 bp); GCLM, 5′-TTTGGTCAGGGAGTTTCCAG-3′, and 5′-TGGTTTTACCTGTGCCCACT-3′ (367 bp); HO-1, 5′-ACATCTATGTGGCCCTGGAG-3′, and 5′-TGTTGGGGAAGGTGAAGAAG-3′ (348 bp); NQO1, 5′-CATTCTGAAAGGCTGGTTTGA-3′, and 5′-TTTCTTCCATCCTTCCAGGAT-3′ (298 bp); Nrf2, 5′-CGGTATGCAACAGGACATTG-3′, and 5′-ACTGGTTGGGGTCTTCTGTG-3′ (263 bp); p21/CDKN1A, 5′-GAGCGATGGAACTTCGACTT-3′, and 5′-CAGGTCCACATGGTCTTCCT-3′ (201 bp); p62/SQSTM1, 5′-GTGGTAGGAACCCGCTACAA-3′, and 5′-CACACTCTCCCCAACGTTCT-3′ (330 bp); PPIA, 5′-GACCCAACACAAATGGTTCC-3′, and 5′-TCGAGTTGTCCACAGTCAGC-3′ (184 bp). Amplified products were electrophoretically resolved by a 2.0% agarose gel, stained with ethidium bromide, and photographed under ultraviolet light.

### Quantitative Reverse Transcriptase-PCR (qRT-PCR)

Wild type mice at 8 weeks of age (male, C57BL/6N) were administered per os with either vehicle (physiological saline) or WN1316 (100 µg/kg body weight). After 1 h, 3 h, 6 h and 9 h, mice were transcardially perfused with physiological saline containing 10% heparin under anesthesia with 2% isoflurane by inhalation, and the lumbar spinal cord was dissected. Total RNA was extracted from lumbar spinal cord using Sepasol-RNA I Super G (Nacalai Tesque), and purified by SV Total RNA Isolation System (Promega) according to manufacturer's instructions. qRT-PCR was performed with QuantiFast™ SYBR Green RT-PCR Kit (QIAGEN) on CFX96 (BIO RAD). The PCR primers (QuantiTect Primer Assays) for HO-1 (Mm_Hmox1_1_SG) and PPIA (Mm_Ppia_1_SG) were purchased from QIAGEN. The levels of all transcripts were normalized for the PPIA mRNA level in each sample. Relative expression was calculated using the ΔΔCt method (Livak 2001).

### RNA Interference

Expression of Nrf2 was inhibited with Hs_NFE2L2_7 HP Validated FlexiTube siRNA (QIAGEN). Silencer Negative Control #1 (NC) siRNA was used as a non-targeting siRNA (AM4635, Ambion). SH-SY5Y cells at 50–60% confluence were transfected with NC and Nrf2 siRNA (5 nM) using Lipofectamine RNAiMAX (Invitrogen). After 24 h and 48 h, cells were subjected to the isolation of RNA and protein, respectively. The degree of Nrf2 expression inhibition was evaluated by RT-PCR and Western blotting.

### Antioxidant Treatment

Differentiated SH-SY5Y cells (0.75×10^4^ cells/well) were treated with 10 µM WN1316 or DMSO in the presence or absence of *N*-acetyl cysteine (NAC) (1 to 2.5 mM) or apocynin (20 to 80 µM) for 3 h (for RT-PCR) or 8 h (for Western blotting and cell viability analysis).

Human monocytic leukemia cell line THP-1 (ATCC, TIB-202) was cultured in RPMI-1640 (Wako) supplemented with 10% fetal bovine serum, 100 U/ml penicillin, and 100 µg/ml streptomycin at 37°C in 5% CO_2_. Approximately 1×10^5^ cells, which were pretreated with 1 to 16 µM WN1316 or DMSO in the presence or absence of 1 mM NAC for 24 h, were plated on a 96-well plate and exposed to 80 µM menadione for 16 h. Cell viability was measured by AlamarBlue assay. For Western blotting, cell lysates were prepared by using M-PER mammalian protein extraction reagent (Thermo scientific) according to standard procedure.

### Measurement of Intracellular Oxidized and Reduced Glutathione

Differentiated SH-SY5Y cells were treated with various concentrations of WN1316 (1–20 µM) or vehicle control (DMSO). After 8 h incubation, the cellular oxidized (GSSG) and reduced glutathione (GSH) levels were measured using the GSH/GSSG-Glo™ Assay kit (Promega) according to the manufacturer’s instructions. GSH and GSSG levels were normalized to protein concentrations.

### Animals

Transgenic mice carrying the H46R mutation in the human *SOD1* gene; SOD1^H46R^ were used as a familial ALS mouse model. The ALS(SOD1^H46R^) transgenic mouse exhibits a slow disease progression with widespread axonal degeneration in the spinal cord when compared with ALS(SOD1^G93A^) transgenic mice (Sasaki 2007, Tanaka 2011, 2008). Since the genetic background is one of the important factors modulating disease phenotypes in mutant *SOD1* transgenic mice, we first generated a congenic line of ALS(SOD1^H46R^) transgenic mice by backcrossing more than 15 generations to B6 mice.

In addition, congenic ALS(SOD1^G93A^) transgenic mice on a B6 background were also generated by crossing B6SJL-TgN(SOD1-G93A)1Gur [ALS(SOD1^G93A^)] males (Jackson Laboratory) to B6 females for more than 11 generations. Both congenic ALS mice were used throughout this study.

The lines were maintained as hemizygotes by mating ALS(SOD1^H46R^) and ALS(SOD1^G93A^) males with B6 females. Offspring were genotyped by PCR using genomic DNA from tail tissue. Mice were housed at 23°C with a 12 h light/dark cycle, in which water and food was available *ad libitum*. The condition of each animal was monitored weekly initially and then daily after disease onset. The lifespan was determined by the days from disease onset to the endpoint, in which mouse lost active and/or spontaneous movement. All animal experimental procedures were approved by the Institutional Animal Care and Use Committee at Tokai University.

### Pharmacokinetics of WN-compounds in Mice

Wild type mice at 8 weeks (male, C57BL/6N) were used for the analysis of CPN-9, WN1303R, WN1312, WN1315 and WN1316 pharmacokinetics. CPN-9 was suspended in 0.5% carboxymethyl cellulose (CMC)-Na (carmellose sodium, Maruishi Pharmaceutical Co., Ltd.), while WN1303R, WN1312 and WN1316 were dissolved in Milli-Q purified water (MQ water) and WN1315 was dissolved in 0.25 N HCl. CPN-9 (100 mg/kg body weight) and the four WN-compounds (50 mg/kg body weight) were injected intragastrically (i.g.). After 15 min, 30 min, 1 h, 2 h, 4 h, 6 h, and 8 h, blood was collected from heart of each mouse anesthetized with 2% isoflurane by inhalation or 4% halothane in a mixture of N_2_O/O_2_ (70∶30). Mice were thereafter perfused transcardially with physiological saline containing 10% heparin under anesthesia, and the whole brain was removed. Each blood sample was centrifuged at 1,000×g for 10 min at 4°C, and the serum fraction was collected. The brain was frozen in liquid nitrogen, and was cryopreserved until use.

We mixed serum with deproteinization buffer (0.05 M H_2_SO_4_, 2.5 M K_2_CO_3_, and diethylester), and collected the organic layer after centrifugation at 1,500×g for 10 min. The organic layer was mixed with 0.05 M H_2_SO_4_, and then, after centrifugation at 1,500×g for 10 min, the water layer was collected. After the addition of methanol, each serum derived sample was subjected to HPLC.

Mouse whole brain was homogenized with acetonitrile and left at −80°C for 10 min. The supernatant was collected after centrifugation at 10,000×g for 10 min at 4°C and was dried under vacuum. The dried sample was re-dissolved in methanol and mixed with two volumes of 10 mM phosphate buffer (pH7.0), after which the supernatant was collected by centrifugation at 10,000×g for 10 min at 4°C and then left at −80°C for 10 min. To further remove debris, the supernatant was filtered (0.45 µm filter), centrifuged (at 10,000×g for 10 min at 4°C), filtered (0.22 µm filter) and finally centrifuged again. Each sample solution from brain was subjected to HPLC.

The concentrations of CPN-9 and WN1316 in the serum and brain samples were determined by HPLC on a CAPCELL PAK C18 column (SHISEIDO) with 50% acetonitrile (CPN-9), 40% acetonitrile (WN1316) or 45% acetonitrile (WN1303R, WN1312, and WN1315) in 10 mM phosphate buffer (pH 7.0).

### Administration of the Compound

WN1316 was dissolved in MQ water, and diluted with physiological saline. ALS(SOD1^H46R^) transgenic mice were given WN1316 via gastric tube (CAT.No.4202, Fuchigami) at a dose of either 1 µg, 10 µg or 100 µg/5 ml/kg body weight. ALS(SOD1^G93A^) transgenic mice were given 10 µg/kg WN1316. Animals simultaneously treated with physiological saline (vehicle group, 5 ml/kg) were used as controls. We commenced daily compound administration when mice showed signs of motor dysfunction (deemed age of onset) between 121d and 130d in ALS(SOD1^H46R^) transgenic mice and between 88d and 106d in ALS(SOD1^G93A^) transgenic mice, and continued until their endpoint.

### Assessment of the Onset of the Symptom

To determine the onset of motor dysfunction in ALS(SOD1^H46R^) transgenic mice, we adopted a balance beam test as previously described in detail [10]. To determine the onset of motor dysfunction in ALS(SOD1^G93A^) transgenic mice, we adopted both balance beam and grip strength tests. Grip strength test was assessed using a traction meter (BrainScience Idea Co., Ltd.). Each mouse was given three trials, and the highest limb strength score was recorded. For ALS(SOD1^G93A^) transgenic mice, grip strength tests were started at 11 weeks of age and performed twice a week until an over 20% reduction compared with the initial limb strength score at 11 weeks of age was observed. Finally, a grade 3 in the balance beam test [10] and over 20% reduction of limb strength score were defined as the sign of the disease onset in ALS(SOD1^G93A^) transgenic mice.

### Observation of Gross Phenotypes in Mice

The gross phenotypes were measured blindly. The body weight of each mouse was measured weekly initially and then daily after disease onset. Mice were assessed for gross behavior every week from 12 weeks of age in ALS(SOD1^H46R^) mice and from 11 weeks of age in ALS(SOD1^G93A^) mice until the endpoint. In particular, hind limb movement and rearing behavior of each animal were monitored weekly from 12 weeks of age until the end stage. The days from the disease onset to the endpoint were counted individually as the survival interval.

### Assessment of Motor Function

We assessed motor performance and coordination, and balance in the mice by the balance-beam test, vertical pole test, and footprint analysis as previously described in detail [10] and grip strength test as described above. In brief, the balance-beam test was performed every day after the onset until mice were unable to stay on the bar. Each mouse was given a maximum of five vertical pole trials using the following two criteria; 1) a cutoff of 45cm and 2) the task was terminated when mouse reached the vertical ascending distance of 45 cm. The highest vertical ascending distance was scored. The footprint trial was conducted at 18 and 21 weeks of age in ALS(SOD1^H46R^) mice and at 12, 16, and 20 weeks of age in ALS(SOD1^G93A^) mice. Grip strength test of ALS(SOD1^H46R^) mice was started at 17 weeks of age and performed once a week.

To evaluate the spontaneous motor activities in mice, we conducted cage activity and rearing performance tests by using SUPERMEX with infrared ray sensor monitor (Muromachi Kikai). Both cage activity and rearing performance were monitored for 2 consecutive nights starting at 18 weeks and 21 weeks of age in ALS(SOD1^H46R^) mice and at 13 weeks, 17 weeks and 21 weeks of age in ALS(SOD1^G93A^) mice. The cumulative counts of cage activity and rearing performance for the dark-period (12 h; 19∶00–7∶00) were analyzed. The ALS(SOD1^H46R^) mice that accidentally died or showed unmeasurable scores on vertical pole test, grip strength test and/or stride length at 21–22 weeks of age (late-symptomatic stage) were omitted from the statistical analyses.

### Histopathological Analysis

At 21–22 weeks of age (late-symptomatic stage), mice were anesthetized with 2% isoflurane by inhalation. Anesthetized mice were transcardially perfused with physiological saline containing 10% heparin, followed by 4% paraformaldehyde (PFA) in 0.1 M phosphate buffer (PB) (pH 7.2). Spinal cord was removed, and lumbar segment was dissected.

For the preparation of paraffin-embedding tissue, lumbar segment was post-fixed with the same fixative for 48 h at 4°C and embedded in paraffin. Serial transverse sections (6 µm thickness) of lumbar segment (L4–L5) were sliced and stained with hematoxylin and eosin (H&E) for histopathological evaluation.

For the preparation of frozen-embedding tissue, lumbar segment was post-fixed with 4% PFA in 0.1 M PB (pH 7.2) for 24 h at 4°C, and then processed in 30% sucrose in 0.1 M PB (pH 7.2) containing 0.1% NaN_3_ for 96 h at 4°C (for cryoprotection) followed by frozen-embedding. Ten µm frozen sections were cut on a cryostat and used for fluorescent immunohistochemistry.

### Immunohistochemical Analysis

Immunohistochemical analyses with anti-ChAT, anti-Iba-1, anti-GFAP, anti-MAP2, anti-IL-1β and anti-8-OHdG antibodies were performed. For immunostaining with anti-ChAT antibody, the deparaffinized sections from late-symptomatic mice were pre-treated by autoclaving at 121°C for 5 min in HISTOFINE antigen retrieval solution (pH 9.0) (Nichirei). For immunostaining with anti-Iba-1 antibody, the deparaffinized sections from late-symptomatic mice were pre-treated by autoclaving at 121°C for 10 min in 10 mM citrate buffer (pH 6.0). For immunostaining with anti-GFAP antibody, the deparaffinized sections were used without any pretreatment. The sections were incubated with 0.3% H_2_O_2_ in methanol for 30 min and then with phosphate-buffered saline (PBS) (pH 7.2) containing 0.3% Triton X-100 for 30 min. After the treatment with PBS containing 5% normal goat serum (NGS) (Vector Laboratories)/0.05% Triton X-100 or 5% normal rabbit serum (NRS) (Vector Laboratories)/0.05% Triton X-100 for 1 h at room temperature, the sections were incubated with either anti-GFAP or anti-Iba-1 antibody in PBS containing 1.5% NGS/0.05% Triton X-100 or with anti-ChAT antibody in PBS containing 1.5% NRS/0.05% Triton X-100 overnight at 4°C. The sections were then incubated with HISTOFINE simple stain mouse MAX-PO (R) (Nichirei) or with HISTOFINE simple stain mouse MAX-PO (G) (Nichirei) for 1 hr at room temperature. The sections were visualized using 0.05% 3,3-diaminobenzidine tetrahydrochloride (DAB) (Wako) and 0.015% H_2_O_2_ in 50 mM Tris-HCl (pH 7.5) buffer, and the DAB reaction products were observed under a light microscope (BIOREVO BZ-9000, KEYENCE).

For double-immunostaining using frozen section, sections from late-symptomatic mice were washed with PBS, and treated with PBS containing 3% normal horse serum (NHS) (Vector Laboratories)/0.3% Triton X-100 for 1 h at room temperature. The sections were incubated with anti-IL-1β antibody in PBS containing 0.01% Triton X-100 for 48 h at 4°C, and then were incubated with CF^TM^488A donkey anti-goat IgG (H+L) (1∶400, Biotium) in PBS containing 0.01% Triton X-100 for 1 h at room temperature. Next, the sections were incubated with anti-GFAP, anti-Iba-1 or anti-MAP2 antibody in PBS containing 0.01% Triton X-100 overnight at 4°C, and then were incubated with CF^TM^555 goat anti-rabbit IgG (H+L) (1∶400, Biotium) in PBS containing 0.01% Triton X-100 for 1 h at room temperature. Fluorescent signals were observed under a light microscope (BIOREVO BZ-9000, KEYENCE).

For double-immunostaining of deparaffinized tissue, sections from late-symptomatic mice were pre-treated by autoclaving at 121°C for 10 min in 10 mM citrate buffer (pH 6.0). After the treatment with PBS containing 5%NGS/0.05% Triton X-100, the sections were incubated with anti-8-OHdG and anti-GFAP, anti-Iba-1 or anti-MAP2 antibodies in PBS containing 1.5% NGS/0.05% Triton X-100 overnight at 4°C. The sections were then incubated with CF^TM^555 Goat anti-mouse IgG (H+L) (1∶200, Biotium) and CF^TM^488A Goat anti-rabbit IgG (H+L) (1∶200, Biotium) in PBS containing 1.5% NGS/0.05% Triton X-100 for 1 h at room temperature, coverslipped using glycerol/PBS containing anti-bleaching agent, and observed under a light microscope (BIOREVO BZ-9000, KEYENCE).

### Quantitative Analysis of the Number of Motor Neurons

Sections (6 µm thickness) of L4–L5 lumbar segment from late-symptomatic mice were immunostained with anti-ChAT (see above), a marker of motor neurons, and observed under a light microscope (BIOREVO BZ-9000, KEYENCE). A total of 10 representative images of every tenth serial section throughout L4–L5 segment were analyzed. A size of neuron (cross-sectional area of each soma) was also determined by utilizing BZ-II Analyzer (KEYENCE). ChAT positive neurons with soma sizes larger than 200 µm^2^ in the anterior horn located within ventral half of the gray matter of the spinal cord were defined as motor neurons (see Figure 5D).

### Preparation of Cell Extracts and Tissue Extracts

SH-SY5Y cells (3×10^5^ cells) were washed twice with ice-cold PBS and incubated in 1 ml of ice-cold 10% trichloroacetic acid for 30 min. Cells were centrifuged at 22,000×g for 15 min at 4°C and lysed by pipetting in 80 µl of 9 M urea containing 2% Triton X-100 and 1% dithiothreitol. After the addition of 20 µl of 10% lithium dodecyl sulfate and 2 µl of 2 M Tris base, cell lysates were sonicated until their viscosity disappeared.

Nuclear extracts were prepared using a nuclear extraction kit (Active Motif) according to the manufacturer’s instruction. Protein concentration was quantified by Pierce 660 nm Protein Assay kit (Thermo Fisher Scientific Inc.) using bovine serum albumin as a standard.

At 21–22 weeks of age (late-symptomatic stage), mice were anesthetized with 2% isoflurane by inhalation and transcardially perfused with physiological saline containing 10% heparin, and the lumbar spinal cord was removed. We homogenized tissues in lysis buffer A [50 mM Tris-HCl (pH7.5), 150 mM NaCl, 1% NP-40, Complete Protease Inhibitor Cocktail (Roche), protein phosphatase inhibitor cocktail (Nacalai tesque)]. After centrifugation at 22,000×g for 30 min at 4°C, we collected the resulting supernatant as a NP-40-soluble fraction. The insoluble pellet fraction was washed with lysis buffer A three times, and then suspended with lysis buffer B [50 mM Tris-HCl (pH7.5), 150 mM NaCl, 5% (w/v) sodium dodecyl sulfate (SDS)], sonicated, and left for 30 min at room temperature. After the centrifugation at 22,000×g for 30 min, the supernatant was collected as a NP-40-insoluble/SDS-soluble fraction. We determined protein concentration by the Pierce 660 nm Protein Assay system (Thermo Fisher Scientific).

### Western Blotting

For Western blotting using cell extracts, equal amounts of protein were electrophoretically separated on 5–20% SDS-polyacrylamide gel (ATTO) and transferred onto polyvinylidene difluoride (PVDF) membrane (Bio-Rad Laboratories). Membrane was blocked with Blocking-One reagent (Nacalai Tesque) for 1 h at room temperature and then incubated with the appropriate primary antibody in Can Get Signal Solution 1 (TOYOBO) overnight at 4°C. The membrane was washed with TBST [20 mM Tris-HCl (pH 7.5), 150 mM NaCl, 0.1% (w/v) Tween-20] three times, and incubated with horseradish peroxidase-conjugated secondary antibody in Can Get Signal Solution 2 (TOYOBO) for 1 h at room temperature. After washing with TBST, signals were visualized by Immobilon Western HRP Substrate (Millipore).

For Western blotting using tissue extracts, protein samples (2 µg) were electrophoretically separated on 5–20% SDS-polyacrylamide gel (ATTO) and transferred on to a PVDF membrane. The membrane was blocked with TBST containing 5% skimmed-milk for 1 h at room temperature and then incubated with the appropriate primary antibody in TBST containing 1% skimmed-milk overnight at 4°C. After washing with TBST buffer, the membrane was incubated with the peroxidase-conjugated secondary antibody in TBST containing 1% skimmed-milk for 1 h at room temperature. After washing with TBST, signals were visualized by Immobilon Western HRP substrate (Millipore).

### Ventral Root Analysis

Under anesthesia with 2% isoflurane by inhalation, mice were transcardially perfused with physiological saline containing 10 U/ml heparin, followed by 2% PFA/2% glutaraldehyde (GA) in 0.1 M PB (pH 7.2). Spinal cord was removed, and L5 ventral root was dissected and fixed with the same fixative for 48 h at 4°C and with 2.5% GA for 4 h at 4°C, followed by washing with 0.1 M PB (pH 7.2), and then post-fixed with 1% osmium tetroxide in 0.05 M PB (pH 7.2) for 2 h at 4°C. After dehydration in graded alcohol and in acetone, the tissues were embedded in epoxy resin. Semi-thin sections (2 µm thickness) of L5 ventral root were stained with 0.5% toluidine blue. The number of myelinated axons was observed under a light microscope (BIOREVO BZ-9000, KEYENCE). Axons over 4 µm in diameter per whole L5 ventral root were counted by utilizing BZ-II Analyzer (KEYENCE).

### Morphological Analysis

Mice were euthanized by cervical dislocation after anesthesia with 2% isoflurane by inhalation. Fresh mouse muscle (quadriceps femoris) at a late-symptomatic stage (21–22 weeks of age) was harvested and flash frozen on 2-methylbutane cooled in liquid nitrogen. Transverse 10 µm sections were processed H&E and NADH histochemistry according to standard procedures.

### Statistical Analysis

Data in this study were expressed as mean ± SD or mean ± SEM. Statistical analyses were conducted using PRISM 5 (GraphPad). Statistical significance was evaluated by ANOVA followed by appropriate *post hoc* test for multiple comparisons between groups and by unpaired Student’s *t*-test for comparisons between the data of two groups. Survival data were compared using Kaplan-Meier survival analysis with Log-rank test. We considered *p*-values <0.05 to be statistically significant.

## Supporting Information

Figure S1 **Workflow for** ***in silico*** **screening combined with** ***in vitro*** **drug screening of anti-oxidative stress compounds.** In the first stage, we performed virtual designing of novel molecules based on the chemical structure of CPN-9 we identified previously [14]. For optimization of virtual designed molecules and CNS drugs, we defined the four criteria. According to these criteria, we selected active molecules, synthesized them, and then identified hit compound via the analysis of anti-oxidative stress cell death (AOSCD). In the second stage, we again virtually designed novel molecules based on the chemical structure of hit compound, and synthesized them. The AOSCD analysis was performed to select the promising compounds.
(TIF)

Figure S2 **Effect of WN compounds against various cytotoxins.** Differentiated SH-SY5Y cells were pretreated with 40 µM CPN-9, 10 µM WN1303R, 6 µM WN1312, 30 µM WN1315, 6 µM WN1316 or DMSO for 8 h at 37°C. The appropriate amount of each cytotoxin, which includes free radical generating compounds (α-naphthoquinone, 12.5 µM for 4 h; 6-OHDA, 400 µM for 4 h), protein kinase inhibitor (staurosporine, 25 µM for 6 h), and phosphatase inhibitor (okadaic acid, 1 µM for 6 h), was added, and incubated for another 4 to 6 h. The cell viability was measured by AlamarBlue assay. Data are expressed as mean ± SD (n = 4). *p<0.001 by one-way ANOVA with Dunnett’s *post hoc* test compared with DMSO-treated control.
(TIF)

Figure S3 **NAC decreases the level of WN1316-induced Nrf2-regulated antioxidant proteins.** (A) Effect of WN1316 on nuclear translocation of Nrf2. Differentiated SH-SY5Y cells were pre-incubated with 40 µM CPN-9, 10 µM WN1316, or DMSO for 3 h, and nuclear fractions were prepared using a nuclear extraction kit (Active Motif). The fractions were subjected to Western blot analysis using anti-Nrf2 antibody. Lamin B1 was used as an internal control for nuclear fraction. (B and C) Effect of NAC on the expression of mRNAs and proteins, including Nrf2, ATF3, Keap1, HO-1, NQO1, GCLM, p62 and p21, in WN1316-treated cells. Differentiated SH-SY5Y cells were treated with 10 µM WN1316 or DMSO in the presence or absence of 2.5 mM NAC for 3 h. Expressions of the above-mentioned mRNAs and proteins were analyzed by RT-PCR (B) and Western blotting (C), respectively. PPIA was used as a control for RT-PCR. β-tubulin was used as a loading control for protein. (D) Effect of NAC and apocynin (APO) on the WN1316-mediated anti-oxidative stress activity. Differentiated SH-SY5Y cells were treated with 8 µM WN1316 or DMSO for 8 h in the presence or absence of NAC or APO, and then exposed to 60 µM menadione for 4 h. The cell viability was determined by AlamarBlue. Data are expressed as mean ± SD (n = 4). *p<0.001 by one-way ANOVA with Dunnett’s *post hoc* test compared with WN1316-treated cells.
(TIF)

Figure S4 **WN1316 upregulates GSH level in dose-dependent manner.** (A) Determination of the optimal concentration of WN1316. Differentiated SH-SY5Y cells were treated with the indicated concentration of WN1316 for 8 h, and then treated with 60 µM menadione for 4 h. The cell viability was determined by AlamarBlue. Data are expressed as mean ± SD (n = 4). *p<0.001 by one-way ANOVA with Dunnett’s *post hoc* test compared with DMSO-treated control. (B–C) Concentration-dependent induction of total reduced glutathione (GSH) and oxidized glutathione (GSSG) levels by WN1316 in differentiated SH-SY5Y cells was analyzed. Differentiated SH-SY5Y cells were treated with the indicated concentration of WN1316 for 8 h. Intracellular total GSH (B) and GSSG levels (C) were measured. Data are expressed as mean ± SD (n = 4). *p<0.001 by one-way ANOVA with Dunnett’s *post hoc* test compared with DMSO-treated control. (D) The ratio of GSH and GSSG was calculated according to the manufacturer’s instructions. Data are expressed as mean ± SD (n = 4). *p<0.001 by one-way ANOVA with Dunnett’s *post hoc* test compared with DMSO-treated control.
(TIF)

Figure S5 **Effect of the WN1316 treatment on the gross clinical symptoms in ALS(SOD1^G93A^) mice.** (A) Changes in the body weight of female and male ALS(SOD1^G93A^) mice in vehicle (female, n = 7) and 10 µg/kg WN1316 (female, n = 8)-treated groups between 8 and 23 weeks of age. Data are expressed as mean ± SD. (B) Scores of the balance beam test. Duration of date from the onset to the day at which each mouse was unable to stay on the bar is shown as Box-whisker plots. Data are expressed as mean ± SEM [vehicle, 39.3±2.1 days (n = 7), and 10 µg/kg WN1316, 47.1±3.0 days (n = 8)]. (C) Footprints of vehicle and 10 µg/kg WN1316-treated mice at 12, 16 and 20 weeks of age. Blue, front paws; red, hind paws. (D) Box-whisker plots of stride length. Data indicate the average distance between the hind paw steps in vehicle (n = 7) and 10 µg/kg WN1316 (n = 8)-treated mice at 12, 16 and 20 weeks of age. *** *p*<0.01 by Student’s *t*-test. (E) The cage activity and (F) rearing performance of vehicle and 10 µg/kg WN1316-treated mice at 13, 17 and 21 weeks of age. Cumulative data counting for 2 consecutive nights are shown as Box-whisker plots (AU; arbitrary unit, each group; n = 5). (G) Effect of the WN1316 treatment on the survival in ALS(SOD1^G93A^) mice. The Kaplan-Meier curves demonstrate the probability of survival interval of vehicle control and 10 µg/kg WN1316-treated ALS(SOD1^G93A^) mice. The average onset of ALS(SOD1^G93A^) mice was 97.7±5.3 days (n = 15). Survival interval in WN1316-treated group (66.1±12.0 days, n = 8) was significantly longer than that in vehicle group (57.9±6.0 days, n = 7) (*p*<0.05 by log-rank test). These data are expressed as mean ± SD.
(TIF)

Figure S6 **The WN1316 treatment does not affect the expression of the mutant SOD1.** The expression levels of the SOD1 protein in the lumbar spinal cord from ALS(SOD1^H46R^) mice treated with vehicle or 10 µg/kg WN1316 at a late symptomatic stage (21–22 weeks of age) and from age-matched non-Tg littermates were analyzed. The NP-40 soluble (A) and NP-40 insoluble/SDS soluble (B) fractions (2 µg proteins each) were used for immunoblotting with anti-SOD1 antibody. hSOD1 represents the mutated (H46R) human SOD1 protein. Glyceraldhyde-3-phosphate dehydrogenase (GAPDH) and β-actin were used as an internal control. Quantitative analyses of SOD1 protein from vehicle-treated and 10 µg/kg WN1316-treated ALS(SOD1^H46R^) mice in NP-40 soluble fraction (C) and NP-40 insoluble/SDS soluble fraction (D) are shown. Data are expressed as mean ± SEM (n = 4).
(TIF)

Figure S7 **Potential mechanism of WN1316-mediated neuroprotection against oxidative stress-induced cell death.** *In vitro* pathway, WN1316 exerts neuroprotective potency against oxidative stress-induced cell death via the upregulation of endogenous NAIP and the activation of Nrf2 signaling cascade in an intracellular ROS-dependent manner and in a positive feedback manner between Nrf2-Keap1 complex and p62/p21. *In vivo* pathway, post-onset administration of WN1316 to ALS mouse models slows disease progression via the suppression of glial activation and neuronal inflammation by inhibiting the generation of inflammatory factor, the reduction of oxidative damage, and the protection of motor neurons and ventral motor axons.
(TIF)
