# Supplementary figures and images for: A Novel Acylaminoimidazole Derivative, WN1316, Alleviates Disease Progression via Suppression of Glial Inflammation in ALS Mouse Model
Source: PLoS One. 2014 Jan 31;9(1):e87728. doi: 10.1371/journal.pone.0087728 (PMC3909264; doi:10.1371/journal.pone.0087728)

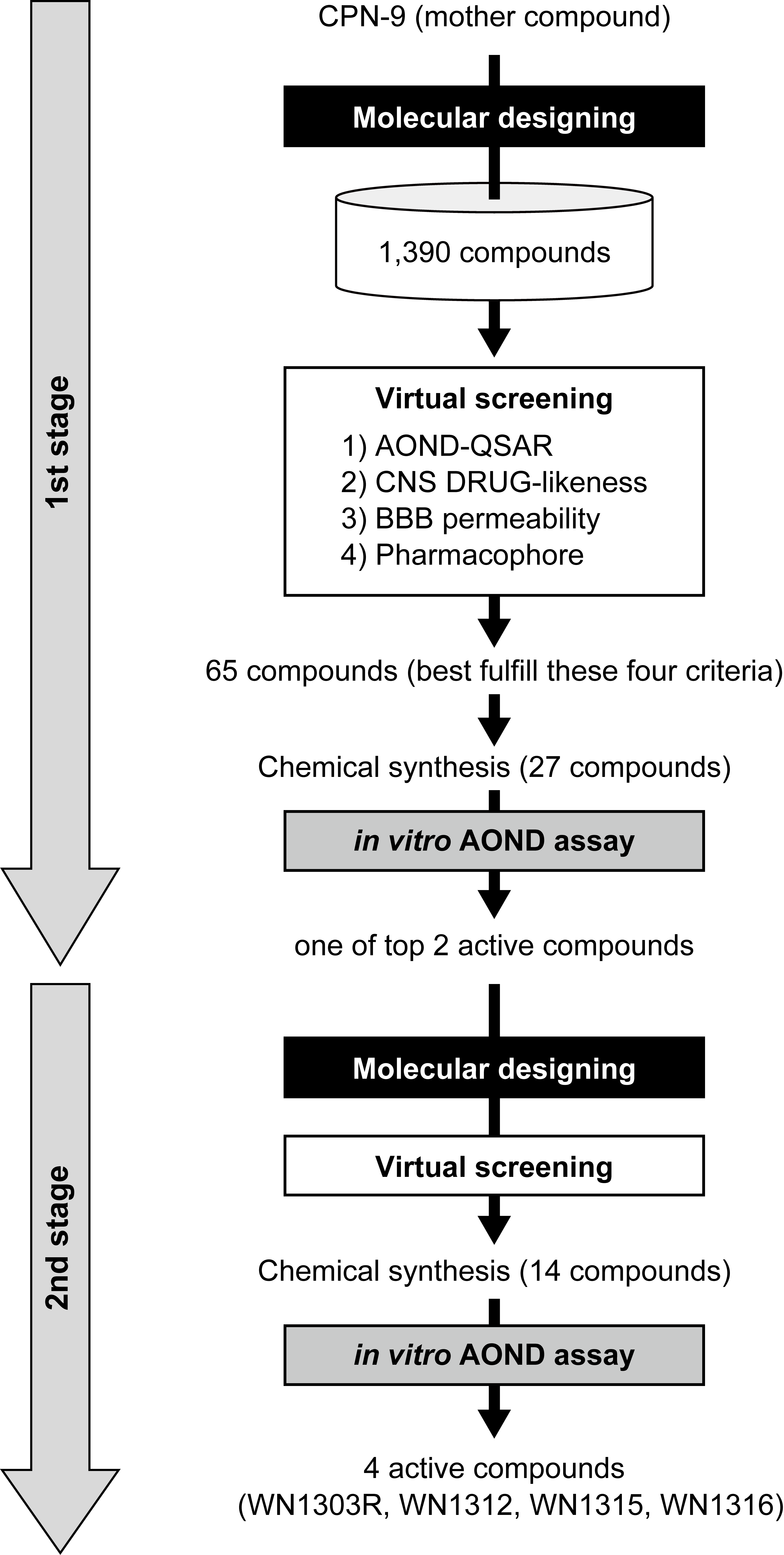

Supplement: Figure S1 — Workflow for in silico screening combined with in vitro drug screening of anti-oxidative stress compounds. In the first stage, we performed virtual designing of novel molecules based on the chemical structure of CPN-9 we identified previously [14]. For optimization of virtual designed molecules and CNS drugs, we defined the four criteria. According to these criteria, we selected active molecules, synthesized them, and then identified hit compound via the analysis of anti-oxidative stress cell death (AOSCD). In the second stage, we again virtually designed novel molecules based on the chemical structure of hit compound, and synthesized them. The AOSCD analysis was performed to select the promising compounds. (TIF) [file pone.0087728.s001.tif]

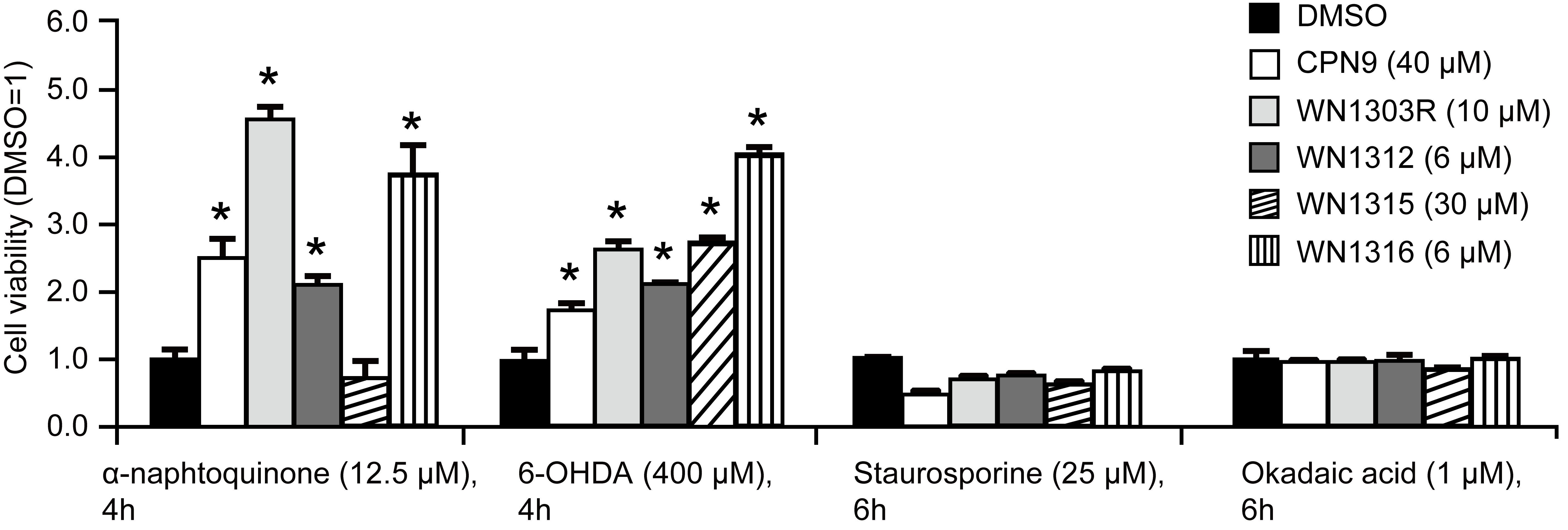

Supplement: Figure S2 — Effect of WN compounds against various cytotoxins. Differentiated SH-SY5Y cells were pretreated with 40 µM CPN-9, 10 µM WN1303R, 6 µM WN1312, 30 µM WN1315, 6 µM WN1316 or DMSO for 8 h at 37°C. The appropriate amount of each cytotoxin, which includes free radical generating compounds (α-naphthoquinone, 12.5 µM for 4 h; 6-OHDA, 400 µM for 4 h), protein kinase inhibitor (staurosporine, 25 µM for 6 h), and phosphatase inhibitor (okadaic acid, 1 µM for 6 h), was added, and incubated for another 4 to 6 h. The cell viability was measured by AlamarBlue assay. Data are expressed as mean ± SD (n = 4). *p<0.001 by one-way ANOVA with Dunnett’s post hoc test compared with DMSO-treated control. (TIF) [file pone.0087728.s002.tif]

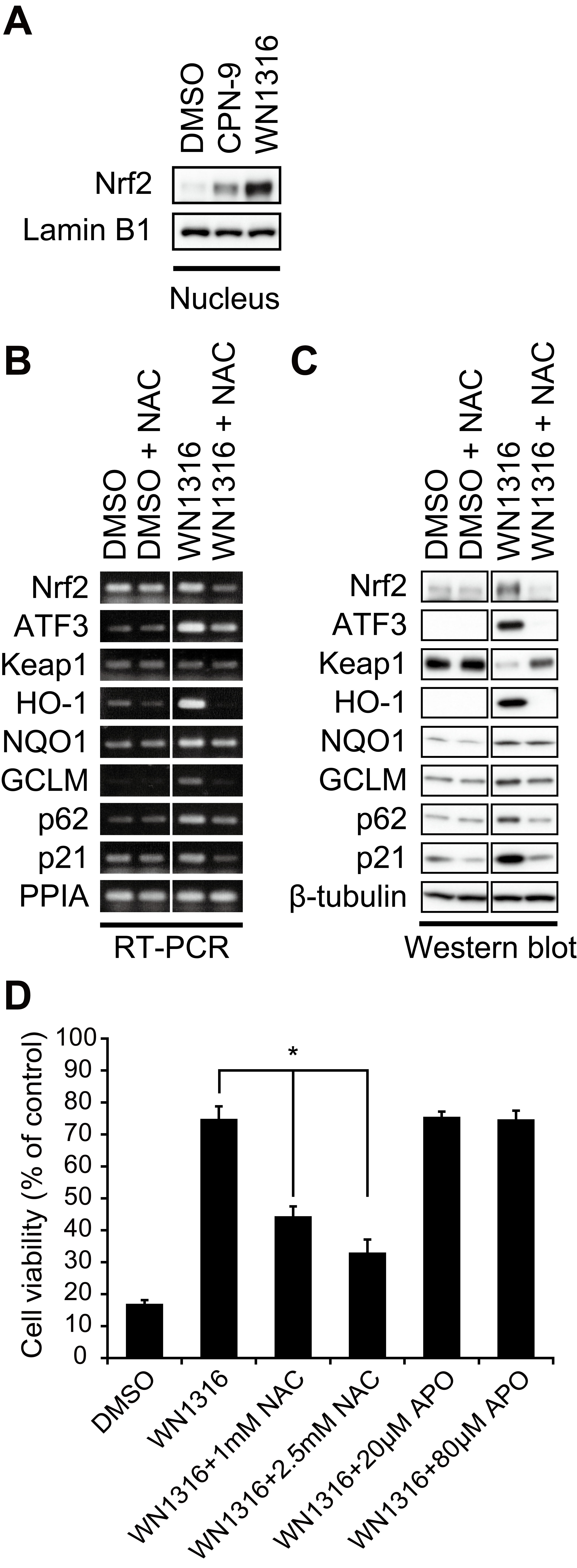

Supplement: Figure S3 — NAC decreases the level of WN1316-induced Nrf2-regulated antioxidant proteins. (A) Effect of WN1316 on nuclear translocation of Nrf2. Differentiated SH-SY5Y cells were pre-incubated with 40 µM CPN-9, 10 µM WN1316, or DMSO for 3 h, and nuclear fractions were prepared using a nuclear extraction kit (Active Motif). The fractions were subjected to Western blot analysis using anti-Nrf2 antibody. Lamin B1 was used as an internal control for nuclear fraction. (B and C) Effect of NAC on the expression of mRNAs and proteins, including Nrf2, ATF3, Keap1, HO-1, NQO1, GCLM, p62 and p21, in WN1316-treated cells. Differentiated SH-SY5Y cells were treated with 10 µM WN1316 or DMSO in the presence or absence of 2.5 mM NAC for 3 h. Expressions of the above-mentioned mRNAs and proteins were analyzed by RT-PCR (B) and Western blotting (C), respectively. PPIA was used as a control for RT-PCR. β-tubulin was used as a loading control for protein. (D) Effect of NAC and apocynin (APO) on the WN1316-mediated anti-oxidative stress activity. Differentiated SH-SY5Y cells were treated with 8 µM WN1316 or DMSO for 8 h in the presence or absence of NAC or APO, and then exposed to 60 µM menadione for 4 h. The cell viability was determined by AlamarBlue. Data are expressed as mean ± SD (n = 4). *p<0.001 by one-way ANOVA with Dunnett’s post hoc test compared with WN1316-treated cells. (TIF) [file pone.0087728.s003.tif]

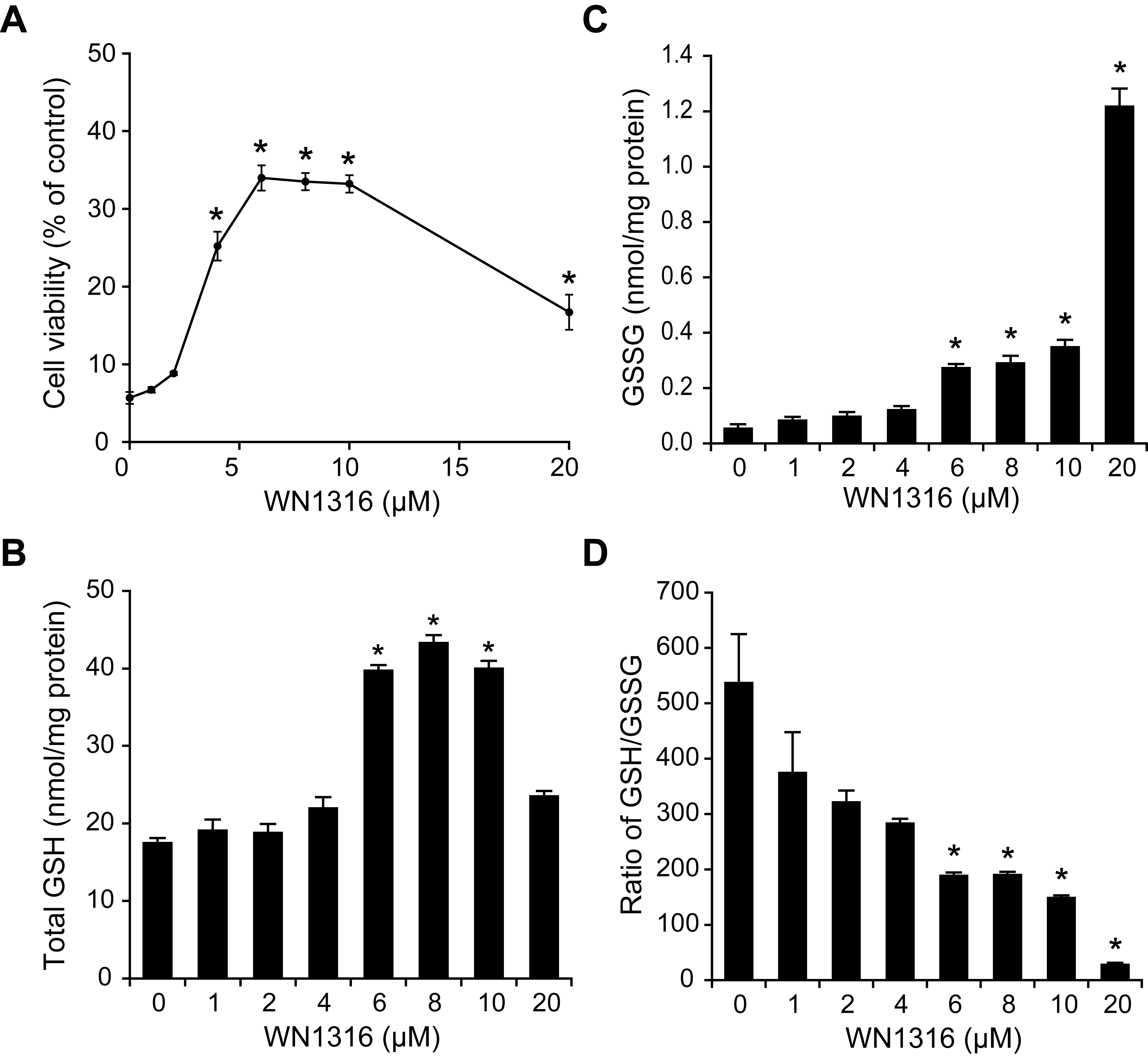

Supplement: Figure S4 — WN1316 upregulates GSH level in dose-dependent manner. (A) Determination of the optimal concentration of WN1316. Differentiated SH-SY5Y cells were treated with the indicated concentration of WN1316 for 8 h, and then treated with 60 µM menadione for 4 h. The cell viability was determined by AlamarBlue. Data are expressed as mean ± SD (n = 4). *p<0.001 by one-way ANOVA with Dunnett’s post hoc test compared with DMSO-treated control. (B–C) Concentration-dependent induction of total reduced glutathione (GSH) and oxidized glutathione (GSSG) levels by WN1316 in differentiated SH-SY5Y cells was analyzed. Differentiated SH-SY5Y cells were treated with the indicated concentration of WN1316 for 8 h. Intracellular total GSH (B) and GSSG levels (C) were measured. Data are expressed as mean ± SD (n = 4). *p<0.001 by one-way ANOVA with Dunnett’s post hoc test compared with DMSO-treated control. (D) The ratio of GSH and GSSG was calculated according to the manufacturer’s instructions. Data are expressed as mean ± SD (n = 4). *p<0.001 by one-way ANOVA with Dunnett’s post hoc test compared with DMSO-treated control. (TIF) [file pone.0087728.s004.tif]

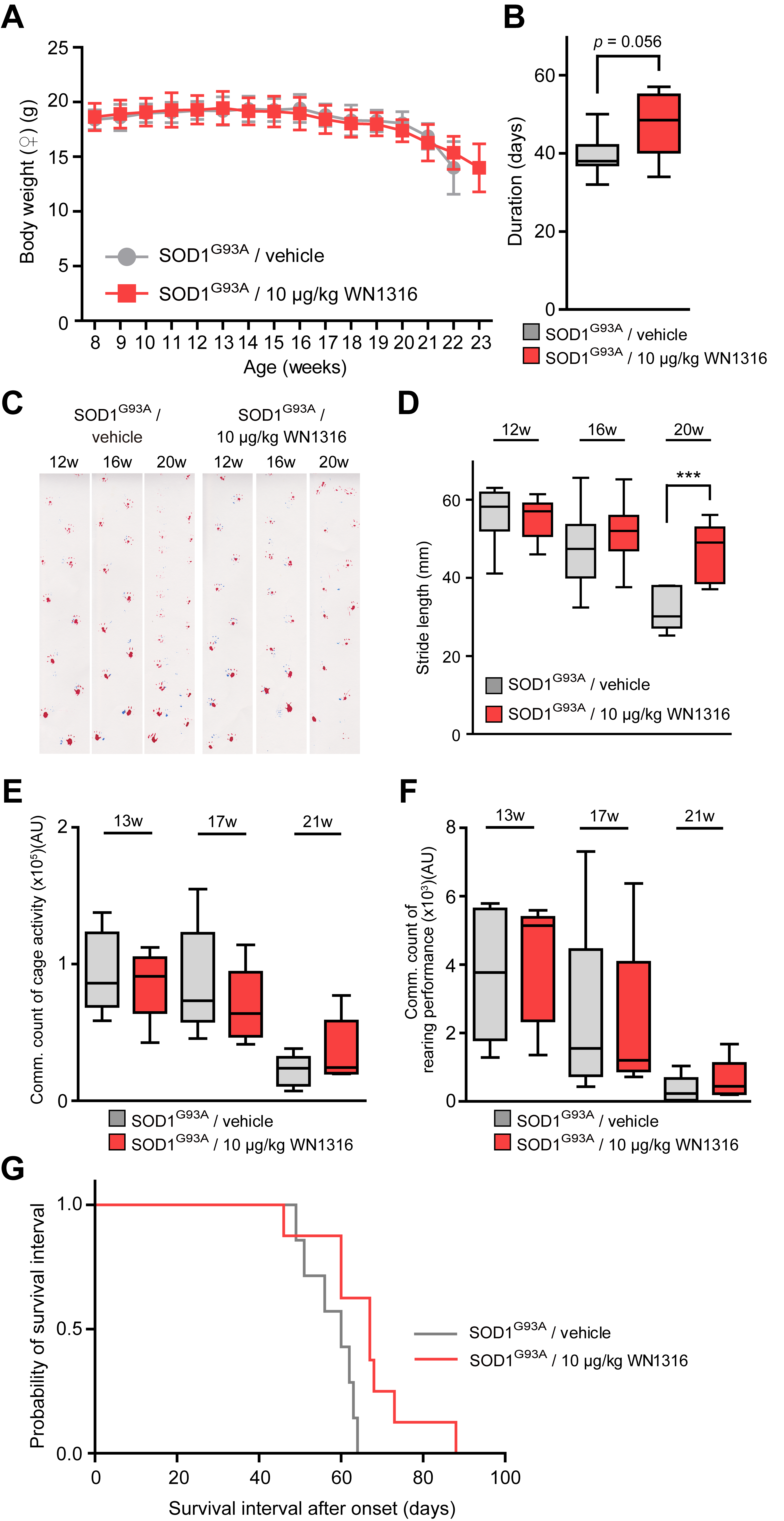

Supplement: Figure S5 — Effect of the WN1316 treatment on the gross clinical symptoms in ALS(SOD1G93A) mice. (A) Changes in the body weight of female and male ALS(SOD1G93A) mice in vehicle (female, n = 7) and 10 µg/kg WN1316 (female, n = 8)-treated groups between 8 and 23 weeks of age. Data are expressed as mean ± SD. (B) Scores of the balance beam test. Duration of date from the onset to the day at which each mouse was unable to stay on the bar is shown as Box-whisker plots. Data are expressed as mean ± SEM [vehicle, 39.3±2.1 days (n = 7), and 10 µg/kg WN1316, 47.1±3.0 days (n = 8)]. (C) Footprints of vehicle and 10 µg/kg WN1316-treated mice at 12, 16 and 20 weeks of age. Blue, front paws; red, hind paws. (D) Box-whisker plots of stride length. Data indicate the average distance between the hind paw steps in vehicle (n = 7) and 10 µg/kg WN1316 (n = 8)-treated mice at 12, 16 and 20 weeks of age. *** p<0.01 by Student’s t-test. (E) The cage activity and (F) rearing performance of vehicle and 10 µg/kg WN1316-treated mice at 13, 17 and 21 weeks of age. Cumulative data counting for 2 consecutive nights are shown as Box-whisker plots (AU; arbitrary unit, each group; n = 5). (G) Effect of the WN1316 treatment on the survival in ALS(SOD1G93A) mice. The Kaplan-Meier curves demonstrate the probability of survival interval of vehicle control and 10 µg/kg WN1316-treated ALS(SOD1G93A) mice. The average onset of ALS(SOD1G93A) mice was 97.7±5.3 days (n = 15). Survival interval in WN1316-treated group (66.1±12.0 days, n = 8) was significantly longer than that in vehicle group (57.9±6.0 days, n = 7) (p<0.05 by log-rank test). These data are expressed as mean ± SD. (TIF) [file pone.0087728.s005.tif]

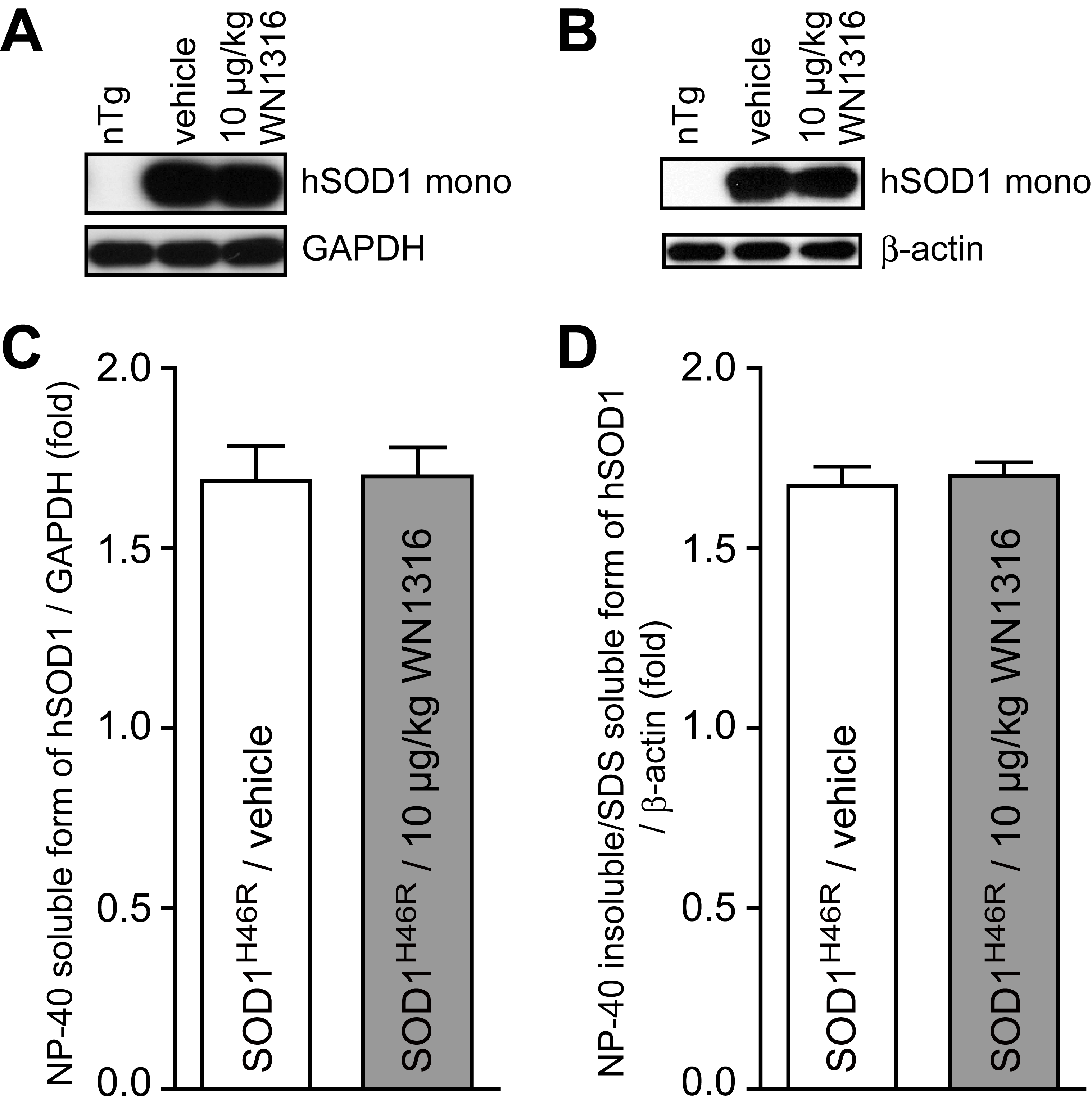

Supplement: Figure S6 — The WN1316 treatment does not affect the expression of the mutant SOD1. The expression levels of the SOD1 protein in the lumbar spinal cord from ALS(SOD1H46R) mice treated with vehicle or 10 µg/kg WN1316 at a late symptomatic stage (21–22 weeks of age) and from age-matched non-Tg littermates were analyzed. The NP-40 soluble (A) and NP-40 insoluble/SDS soluble (B) fractions (2 µg proteins each) were used for immunoblotting with anti-SOD1 antibody. hSOD1 represents the mutated (H46R) human SOD1 protein. Glyceraldhyde-3-phosphate dehydrogenase (GAPDH) and β-actin were used as an internal control. Quantitative analyses of SOD1 protein from vehicle-treated and 10 µg/kg WN1316-treated ALS(SOD1H46R) mice in NP-40 soluble fraction (C) and NP-40 insoluble/SDS soluble fraction (D) are shown. Data are expressed as mean ± SEM (n = 4). (TIF) [file pone.0087728.s006.tif]

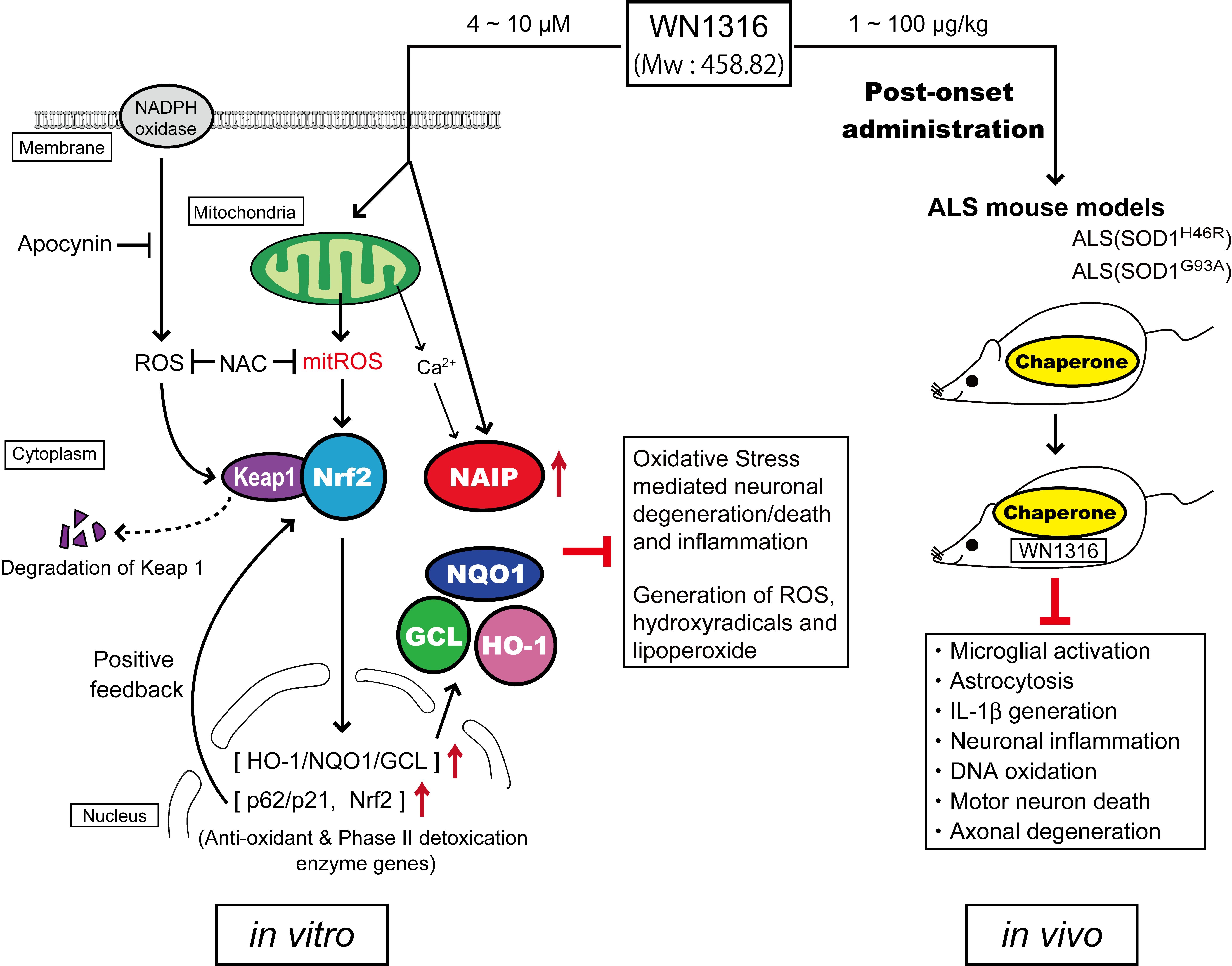

Supplement: Figure S7 — Potential mechanism of WN1316-mediated neuroprotection against oxidative stress-induced cell death. In vitro pathway, WN1316 exerts neuroprotective potency against oxidative stress-induced cell death via the upregulation of endogenous NAIP and the activation of Nrf2 signaling cascade in an intracellular ROS-dependent manner and in a positive feedback manner between Nrf2-Keap1 complex and p62/p21. In vivo pathway, post-onset administration of WN1316 to ALS mouse models slows disease progression via the suppression of glial activation and neuronal inflammation by inhibiting the generation of inflammatory factor, the reduction of oxidative damage, and the protection of motor neurons and ventral motor axons. (TIF) [file pone.0087728.s007.tif]
